# Mapping individual cortico–basal ganglia–thalamo–cortical circuits integrating structural and functional connectome: implications for upper limb motor impairment poststroke

**DOI:** 10.1002/mco2.764

**Published:** 2024-10-06

**Authors:** Xin Xue, Jia‐Jia Wu, Xiang‐Xin Xing, Jie Ma, Jun‐Peng Zhang, Yun‐Ting Xiang, Mou‐Xiong Zheng, Xu‐Yun Hua, Jian‐Guang Xu

**Affiliations:** ^1^ Department of Rehabilitation Medicine Yueyang Hospital of Integrated Traditional Chinese and Western Medicine Shanghai University of Traditional Chinese Medicine Shanghai China; ^2^ Engineering Research Center of Traditional Chinese Medicine Intelligent Rehabilitation Ministry of Education Shanghai China; ^3^ Rehabilitation Center Qilu Hospital of Shandong University Jinan China; ^4^ School of Rehabilitation Science Shanghai University of Traditional Chinese Medicine Shanghai China; ^5^ Department of Traumatology and Orthopedics Yueyang Hospital of Integrated Traditional Chinese and Western Medicine Shanghai University of Traditional Chinese Medicine Shanghai China

**Keywords:** cortico–basal ganglia–thalamo–cortical circuits, motor impairment, stroke

## Abstract

This study investigated alterations in functional connectivity (FC) within cortico–basal ganglia–thalamo–cortical (CBTC) circuits and identified critical connections influencing poststroke motor recovery, offering insights into optimizing brain modulation strategies to address the limitations of traditional single‐target stimulation. We delineated individual‐specific parallel loops of CBTC through probabilistic tracking and voxel connectivity profiles‐based segmentation and calculated FC values in poststroke patients and healthy controls, comparing with conventional atlas‐based FC calculation. Support vector machine (SVM) analysis distinguished poststroke patients from controls. Connectome‐based predictive modeling (CPM) used FC values within CBTC circuits to predict upper limb motor function. Poststroke patients exhibited decreased ipsilesional connectivity within the individual‐specific CBTC circuits. SVM analysis achieved 82.8% accuracy, 76.6% sensitivity, and 89.1% specificity using individual‐specific parallel loops. Additionally, CPM featuring positive connections/all connections significantly predicted Fugl‐Meyer assessment of upper extremity scores. There were no significant differences in the group comparisons of conventional atlas‐based FC values, and the FC values resulted in SVM accuracy of 75.0%, sensitivity of 67.2%, and specificity of 82.8%, with no significant CPM capability. Individual‐specific parallel loops show superior predictive power for assessing upper limb motor function in poststroke patients. Precise mapping of the disease‐related circuits is essential for understanding poststroke brain reorganization.

## Graphical abstract

We delineated individual‐specific parallel cortico–basal ganglia–thalamo–cortical (CBTC) circuits through probabilistic tracking and voxel connectivity profiles‐based segmentation and calculated functional connectivity (FC) values within predefined loops in poststroke patients and healthy controls, comparing with conventional atlas‐based FC calculation. Support vector machine (SVM) analysis distinguished poststroke patients from controls. Connectome‐based predictive modeling (CPM) used FC values within CBTC circuits to predict upper limb motor function.

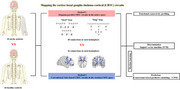

## 1 INTRODUCTION

Motor impairment, a common complication poststroke, significantly diminishes quality of life.^1^, ^2^ While numerous interventions have been developed for motor recovery, their clinical benefits remain limited. Noninvasive brain stimulation, like transcranial magnetic stimulation (TMS), holds promise. The conventional TMS protocol involves low‐frequency rTMS on the unaffected hemisphere's primary motor cortex (M1) and high‐frequency rTMS on the affected M1 cortex, guided by the interhemispheric inhibition model.^3^ However, this approach does not benefit a substantial portion of patients. Recent research has shifted focus from isolated brain regions to distributed networks, emphasizing connectome reorganization in poststroke motor rehabilitation.^4^ Cortico–cortical paired associative stimulation (ccPAS) is a protocol in which repetitive low‐frequency paired stimulation can induce changes in excitability by a spike‐timing dependent plasticity‐like mechanism.^5^ By strategically selecting stimulation sites and optimizing timing intervals, ccPAS promotes the potentiation of interconnected neural pathways crucial for motor function.^6^ Therefore, identifying critical connections influencing functional prognosis has become a top priority.

Cortico–basal ganglia–thalamo–cortical (CBTC) circuits are widely recognized as central to motor control and feedback processes.^7^ Noninvasive imaging techniques, have provided valuable tools for mapping motor‐related circuits in the living human brain.^8^, ^9^ Functional MRI studies have highlighted the role of the motor cortex, basal ganglia, and thalamus in facilitating motor control.^10^, ^11^

To map neural circuits noninvasively in the human brain, techniques include fiber tracking based on diffusion tensor imaging and functional connectivity (FC) analysis, quantifying temporal coherence through correlation analysis of different brain regions using the blood oxygen level‐dependent (BOLD) signal. Standard human brain atlases have been instrumental in these researches. Brain regions parceled according to standard atlases were considered homogeneous structures. However, the subcortical nuclei within the CBTC circuit exhibit complex topographical divisions defined by cortical and subcortical connectivity patterns.^12^ Multimodal MRI, combining diffusion and functional data, offers the potential to bridge brain tract structure with functional networks. Notably, probabilistic diffusion tractography has been used to parcellate subcortical regions, enabling accurate mapping of CBTC circuits at the individual level based on segregated and integrative connectivity patterns in subcortical nuclei.^12^, ^13^, ^14^

In this study, we aimed to investigate FC alterations in CBTC circuits in poststroke patients and identify critical connections impacting functional prognosis. We initially mapped the CBTC circuits using two methods: (1) atlas‐based FC calculation in the standard MNI space, treating each subcortical structure as an unified entity; (2) mapping parallel CBTC circuits in the native space by probabilistic tracking and voxel connectivity profiles (VCPs)‐based segmentation, then calculating FC within predefined CBTC loops. We first compared FC values within CBTC circuits between poststroke patients and healthy controls. To enhance our analysis, we then employed machine learning to identify connections that distinguish healthy controls from poststroke patients and predict clinical motor function. We hypothesized that (1) FC alterations in CBTC circuits significantly differ between poststroke patients and healthy controls, contributing to varied motor dysfunction outcomes; (2) precise mapping of individual‐specific neural circuits is essential for studying diseased‐related brain reorganization, facilitating the development of personalized treatments. By identifying key connections influencing rehabilitation outcomes and optimizing target planning for brain modulation in the connectomics era, we aim to achieve significant clinical benefits through therapeutic brain stimulation.

## 2 RESULTS

### 2.1 Demographic information

**FIGURE 1:**
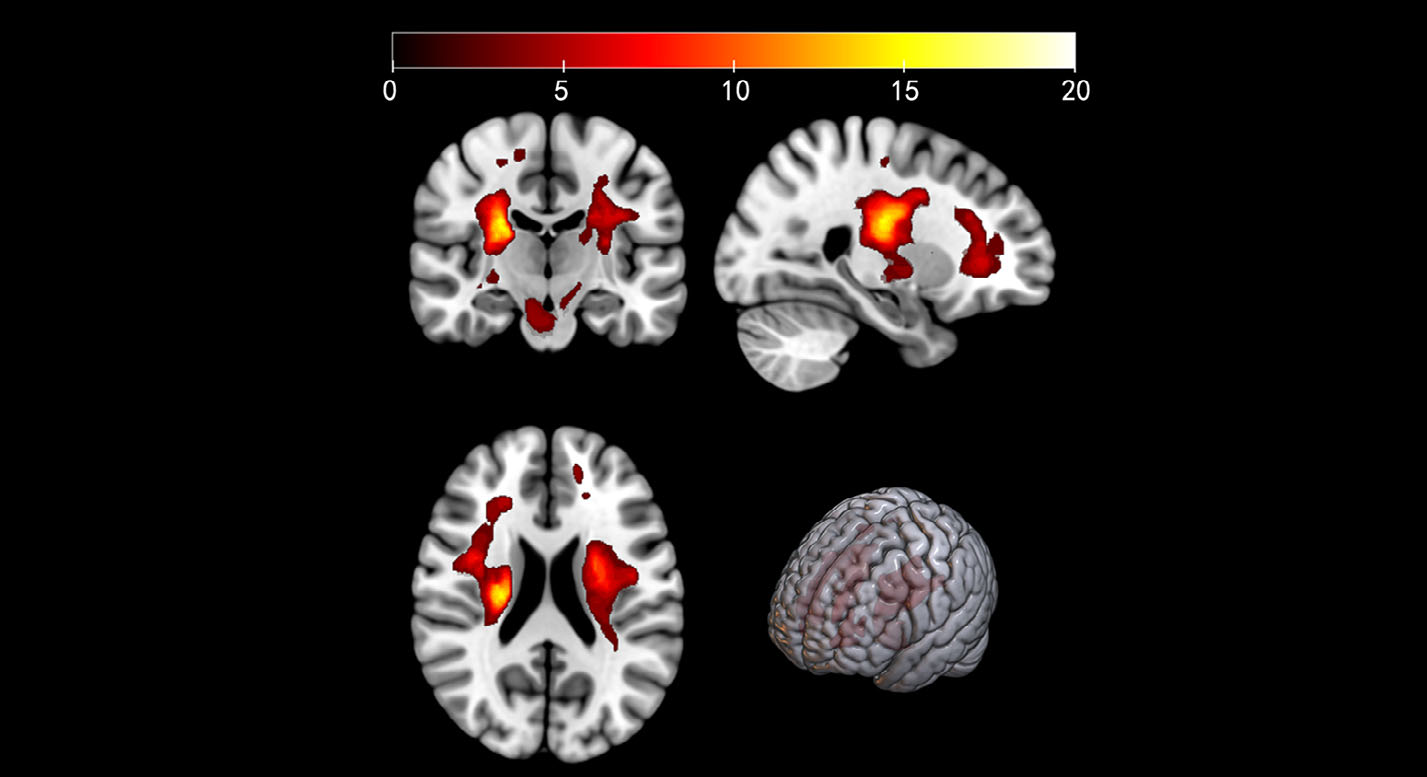
Distribution of stroke lesions in the entire sample of patients. The color scale represents number of participants with lesioned voxel as evaluated by T1 images.

A total of 128 participants were enrolled as the discovery dataset, including 64 stroke patients (35 male and 29 female, age: 59 (18.2) years) and 64 healthy controls (36 male and 28 female, age: 54 (19.5) years). Age was analyzed using the Mann–Whitney *U* test as the distribution was not normal, and gender was analyzed by Chi‐square test. All participants characteristics are shown in Table 1, and the distribution of lesions is shown in Figure 1. Additionally, the detailed information for all patients is presented in Table S1. The results showed no significant differences between groups in age (*z* = −1.004, *p* = 0.316) and gender (*χ* ^2^ = 0.032, *p* = 0.859).

**TABLE 1:** Demographic and clinical characteristics of enrolled participants.

Characteristic	Stroke patients (*n* = 64)	Healthy controls (*n* = 64)	*z*/*χ* ^2^	*p* Value
Age (yr)^*^	59 (18.2)	54 (19.5)	−1.004	0.316
Gender (male/female)^†^	35/29	36/28	0.032	0.859
Diabetes mellitus, *n* (%)^†^	32 (50)	–		–
Hypertension, *n* (%)^†^	46 (71.9)	–		–
Stroke type (ischemic/hemorrhagic)^†^	48/16	–		–
Disease course (month)^*^	7.5 (7.0)	–		–
UE‐FMA^*^	15.5 (26.5)	–		–

Data are expressed as the median (interquartile range, IQR) (*), number (†), or percentage.

Abbreviation: UE‐FMA, The Upper Extremity Fugl‐Meyer Assessment.

### 2.2 Poststroke patients exhibited decreased ipsilesional connectivity within the individual‐specific CBTC circuits

For conventional atlas‐based FC analysis, there is no significant differences between poststroke patients and healthy controls. Figure S1 presents the conventional atlas‐based FC for individual participants within each group, encompassing 26 connections in each hemisphere.

For FC analysis after subcortical connectivity‐based segmentation, poststroke patients showed decreased FC between the thalamus_DLPFC_ and dorsolateral prefrontal cortex (DLPFC) (*p*‐false discovery rate [FDR] = 0.026) in the specific “long” loop, between the caudate_M1_ and M1 (*p*‐FDR < 0.001) and between the putamen_DLPFC_ and DLPFC (*p*‐FDR = 0.033) in the specific “short” loop within the affected hemisphere (Table 2). Subcortical area with subscript referred to the subdivision of the subcortical area connected with the subscript cortical area. The 40 FC values of each participant are displayed in Figure S2.

**TABLE 2:** Group differences in functional connectivity in CBTC circuits based on probabilistic tracking and voxel connectivity profiles‐based segmentation.

	Hemisphere	ROI1	ROI2	*p‐*FDR value
Patients < healthy controls				
“Long” loop	Affected	thalamus_DLPFC_	DLPFC	0.026
“Short” loop	Affected	caudate_M1_	M1	<0.001
“Short” loop	Affected	putamen_DLPFC_	DLPFC	0.033

Subcortical area with subscript referred to the specific subdivision of the subcortical area connected with the subscript cortical area as determined through diffusion white matter fiber tractography.

Abbreviations: DLPFC, dorsolateral prefrontal cortex. M1, primary motor cortex.

### 2.3 Subject‐specific CBTC circuits outperformed conventional atlas‐based CBTC connections in distinguishing stroke patients from healthy controls

**FIGURE 2:**
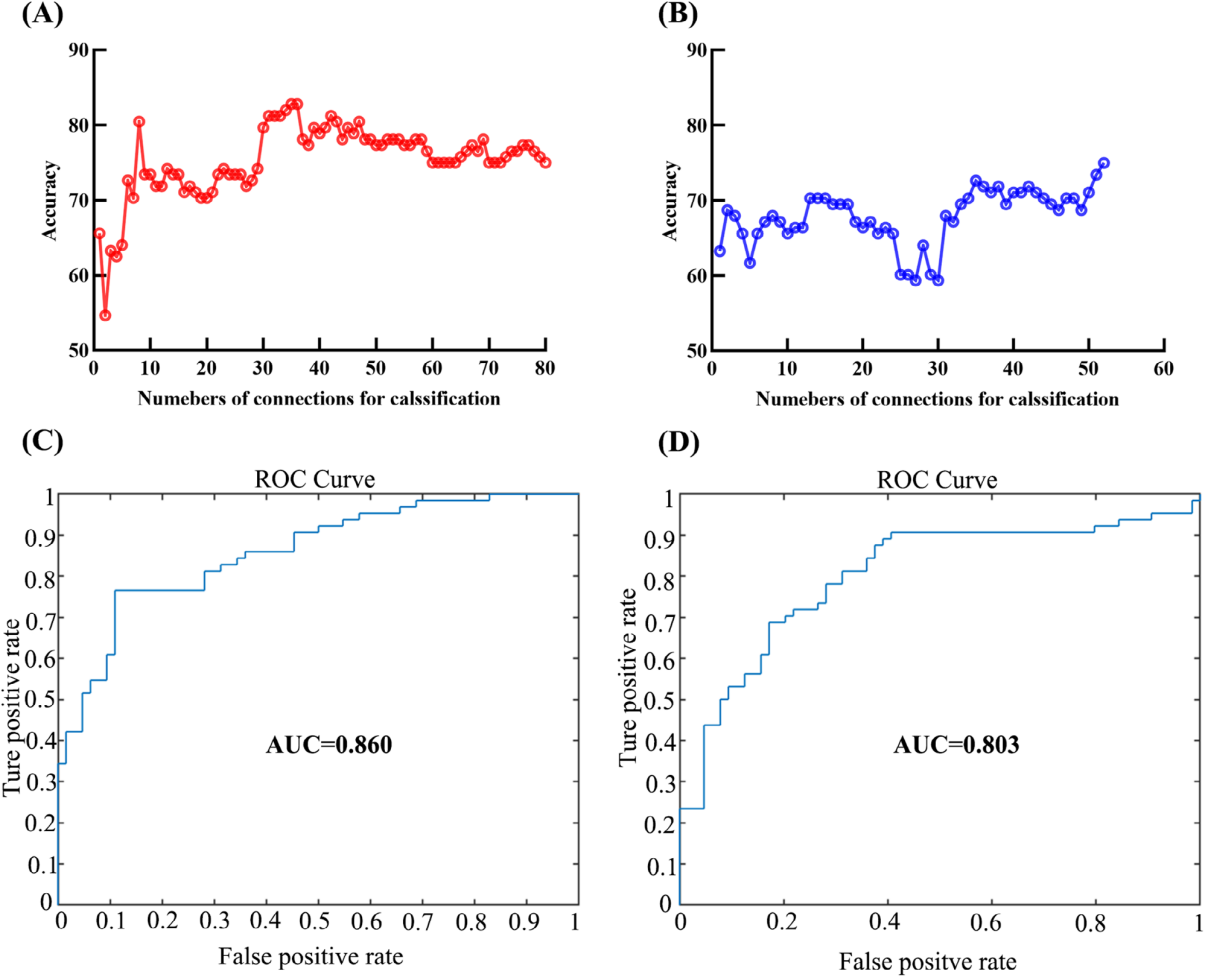
Classification performance of SVM using connections derived from the two CBTC circuits mapping methods. Accuracy as a function of the number of connections used in the classification process for the individual mapping CBTC circuits based on VCP‐based segmentation (A) and for the conventional atlas‐based CBTC circuits (B). The connections were ranked according to *F* scores in descending order. ROC curve of the classifier for the individual mapping CBTC circuits (C) and for the conventional atlas‐based CBTC circuits (D).

The results showed that support vector machine (SVM) classifier using connections derived from individual mapping CBTC circuits within predefined loops after VCPs‐based segmentation performed better than the conventional atlas‐based method. Figure 2A shows that the linear SVM classifier achieved the highest accuracy of 82.8% (*p* < 0.001) with a sensitivity of 76.6%, and a specificity of 89.1% when using the connections of individual mapping CBTC circuits based on VCPs‐based segmentation. As shown in Figure 2C, the ROC curve analysis acquired an area under the curve (AUC) of 0.860, indicating a good classification power.^15^ For the conventional atlas‐based FC analysis, the SVM model correctly classified 75.0% of the participants at most (*p* < 0.001) (Figure 2B), which had an AUC of 0.803 with a sensitivity of 67.2% and a specificity of 82.8% (Figure 2D).

### 2.4 Individual‐specific parallel loops show superior predictive power for assessing upper limb motor function in poststroke patients

**FIGURE 3:**
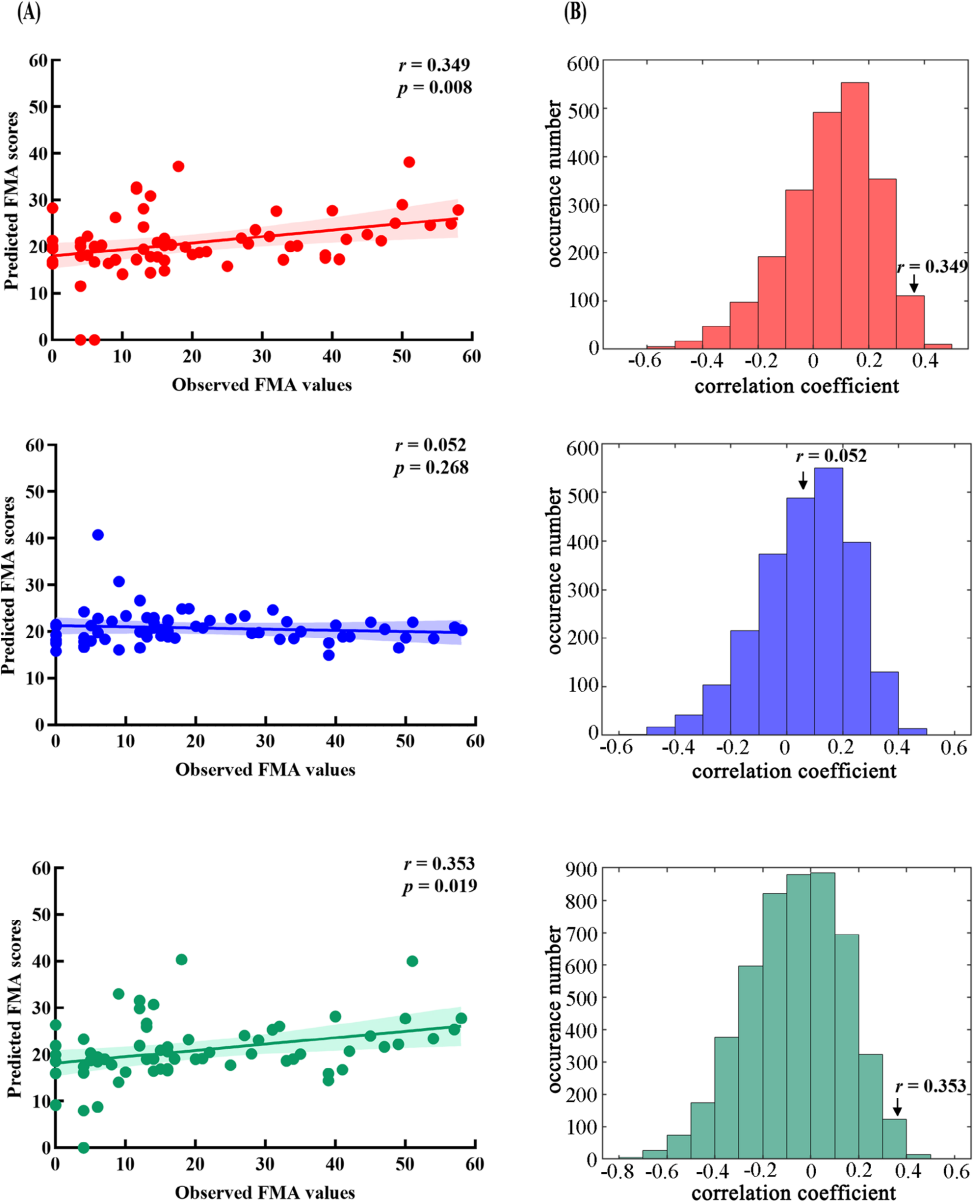
Prediction performance of CPM using connections derived from the individual‐mapping CBTC circuits based on VCP‐based segmentation. (A) Correlation between observed and predicted UE‐FMA scores in positive (red), negative (blue), and combined (green) connections. (B) The distribution of correlation coefficients by a permutation test of 5000 times. *p < 0.05.

The correlation between the observed Fugl‐Meyer Assessment for Upper Extremity (UE‐FMA) scores and predicted UE‐FMA scores represents the predictive efficacy of the connectome‐based predictive modeling (CPM). Results indicated that the proposed individual mapping approach based on VCPs‐based segmentation was better than the conventional atlas‐based methodology in term of predicting motor function. In the individual mapping CBTC circuits, the positive connections (*r* = 0.349, *p* = 0.008) and combined connections (*r *= 0.353, *p* = 0.019) significantly predicted individual difference in UE‐FMA, while negative did not significantly predict individual motor performance (negative connections: *r* = 0.052, *p* = 0.268) (Figure 3). Table 3 show the connections that were most well represented in the positive connections and the combined connections. Specifically, the well‐represented positive connections include the connections between caudate_M1_ and M1 in the “short” loop, putamen_M1_ and M1 in the “short” loop of the affected hemisphere and putamen_DLPFC_ and DLPFC in the “short” loop of the unaffected hemisphere. The well‐represented combined connections build upon these by including the negative connection between the ipsilateral caudate_MPFC_ and medial prefrontal cortex (MPFC) in the “short” loop, which also significantly contributes to the predictive accuracy. The conventional atlas‐based FC values in the CBTC circuits had no significant predictive effect on motor behavioral scores in the CPM model (positive connections: *r* = −0.064, *p* = 0.270; combined connections: *r* = −0.221, *p* = 0.587), and there was no significant negatively correlated connection.

**TABLE 3:** Connections significantly contributing to motor function prediction.

Categories of connections	Hemisphere		Region1	Connected with	Region 2
Positive	Affected	“Short” loop	Caudate_M1_		M1
Positive	Affected	“Short” loop	Putamen_M1_		M1
Positive	Unaffected	“Short” loop	Putamen_DLPFC_		DLPFC
Negative	Unaffected	“Short” loop	Caudate_MPFC_		MPFC

Subcortical area with subscript referred to the specific subdivision of the subcortical area connected with the subscript cortical area as determined through diffusion white matter fiber tractography.

### 2.5 The external cohort validation corroborated the findings observed in the discovery dataset

We next evaluated the classification and predictive efficacy using the same methodology in an independent external validation sample of 62 participants, including 31 stroke patients and 31 age‐ and gender‐matched healthy controls (details in Tables S2 and S3; lesion distribution in Figure S3). Poststroke patients showed decreased FC between the caudate_M1_ and M1 (*p*‐FDR = 0.036) and between the putamen_M1_ and M1 (*p*‐FDR = 0.004) in the “long” loop, and between the putamen_DLPFC_ and DLPFC (*p*‐FDR = 0.036) and between the putamen_M1_ and M1 (*p*‐FDR = 0.036) in the “short” loop within the affected hemisphere, via subcortical connectivity‐based segmentation (Table S4). These differences were not statistically significant for conventional atlas‐based FC. FC values from both methods are shown in Figures S4 and S5. For classification, the linear SVM classifier achieved 95.2% accuracy (*p* < 0.001) with 96.8% sensitivity and 93.6% specificity using the connections from VCPs‐based segmentation (AUC = 0.939). While the conventional atlas‐based FC analysis correctly classified 71.0% of participants (*p *= 0.040) with an AUC of 0.739 (61.3% sensitivity and 80.7% specificity) (Figure S6). Regarding prediction performance, positive connections in individual mapping CBTC circuits significantly predicted UE‐FMA scores (*r* = 0.298, *p* = 0.013), whereas negative and combined connections did not (negative connections: *r* = −0.697, *p* = 0.874; combined connections: *r* = 0.174, *p* = 0.204) (Figure S7). Conventional atlas‐based FC values did not significantly predict motor performance (positive connections: *r* = −0.279, *p* = 0.390; negative connections: *r* = −0.609, *p* = 0.689; combined connections: *r* = 0.105, *p* = 0.265).

### 2.6 The subgroup analysis for ischemic stroke produced results consistent with the initial findings

We then focused on ischemic stroke patients for a reanalysis (discovery dataset, details in Table S1). This sample included 96 participants, with 48 ischemic stroke patients and 48 age‐ and gender‐matched healthy controls. Poststroke patients showed decreased FC between the caudate_M1_ and M1 (*p*‐FDR < 0.001) in the “long” loop and between the caudate_M1_ and M1 (*p*‐FDR = 0.009) in the “short” loop within the affected hemisphere, via subcortical connectivity‐based segmentation (Table S5). These differences were not statistically significant for conventional atlas‐based FC. FC values from both methods are shown in Figures S8 and S9. For classification, the linear SVM classifier achieved 82.3% accuracy (*p* = 0.005) with 75.0% sensitivity and 89.6% specificity using the connections from VCPs‐based segmentation (AUC = 0.868). While the conventional atlas‐based FC analysis correctly classified 78.1% of participants (*p *= 0.002) with an AUC of 0.826 (77.1% sensitivity and 79.1% specificity) (Figure S10). Regarding prediction performance, positive connections in individual mapping CBTC circuits significantly predicted UE‐FMA scores (*r* = 0.180, *p* = 0.045), whereas negative and combined connections did not (negative connections: *r* = −0.856, *p* = 0.998; combined connections: *r* = 0.179, *p* = 0.118) (Figure S11). Conventional atlas‐based FC values did not significantly predict motor performance (positive connections: *r* = 0.025, *p* = 0.141; negative connections: *r* = −0.503, *p* = 0.876; combined connections: *r* = −0.273, *p* = 0.785).

## 3 DISCUSSION

Building upon well‐established methods to map CBTC circuits based on probabilistic tracking and VCPs‐based segmentation,^12^ our study provided a comprehensive analysis of the FC patterns related to poststroke upper motor impairments. To our knowledge, this is the first time that this approach has been used for poststroke patients. The FC values obtained through both methods revealed significant distinctions between stroke patients and the healthy controls, with both methods effectively discriminating stroke patients from the control group. Notably, the FC data derived from subject‐specific CBTC circuit mapping based on probabilistic tracking and VCPs‐based segmentation demonstrated superior accuracy. In our prediction analysis, the connections of subject‐specific CBTC circuits also outperformed the conventional atlas‐based approach in predicting poststroke upper motor function. Moreover, we demonstrate the generalizability of the findings on the ischemic stroke samples and an external dataset.

The journal “Science” featured a series of four consecutive cover articles in 2022, all conveying a unified message: brain functions are not solely confined to isolated brain regions, but rather emerge from the intricate connections and communication between different areas.^16^, ^17^, ^18^, ^19^ This challenges the traditional modular brain concept, which falls short in accounting for interindividual variability. Connectivity‐based approaches enable researchers to model brain specificity in individuals, explore the diversity of brains, and enhance the development of more effective clinical treatments.^20^, ^21^

In past neuroscience research, the intricate connections and circuits existing between the cerebral cortex and basal ganglia have constituted a notably focal area, given their pivotal significance in the regulation of motor functions. FC is an effective index to evaluate motor function recovery and brain plasticity after stroke.^22^, ^23^ It has been established that the abnormal FC patterns exist in the brain networks in stroke patients and are associated with function impairments following stroke.^24^, ^25^ Previous studies predominantly employed atlas‐based methodologies to delineate these connections, including the utilization of anatomical atlases and standardized spatial templates for analysis. Nevertheless, this approach exhibits limitations in elucidating individual differences and structural variations. In line with our results, comparing with healthy controls, the reduced FC was mainly located in the damaged hemisphere. These results indicated that hypofunction of CBTC circuits was associated with motor dysfunction in stroke.

When comparing FC differences between patients and healthy participants, using an approach that calculates FC based on individual CBTC circuits in native space showed significant reductions in connectivity within motor‐related brain regions. In contrast, conventional atlas‐based methods did not reveal pronounced FC differences. This suggests that individual CBTC circuit analysis in native space is more sensitive to detecting connectivity changes in motor‐related areas associated with poststroke motor impairment compared with traditional atlas‐based approaches. Additionally, our results indicated that within classification and prediction models, it showcased enhanced discriminatory ability with healthy subjects and superior performance in predicting behavioral outcomes. Resting‐state fMRI (rs‐fMRI) data have been shown viable in classification and prediction.^26^, ^27^, ^28^, ^29^, ^30^ A reliable neuroimaging‐based classifier can be successfully applied to FC patterns in CBTC circuits using rs‐fMRI data with appropriate feature selection and parameter tuning.^31^ However, in previous classification and prediction studies, the prevalent input features were rooted in atlas‐based whole‐brain FC, with various attempts during the feature selection stage to enhance model accuracy.^32^, ^33^

Diverging from previous data‐driven investigations, this study is oriented toward motor functional rehabilitation and, drawing upon cerebral functional and anatomical underpinnings, it selects neurocircuit connections closely associated with motor functionality as features for classification and prediction, leading to a substantial reduction in redundant features. Furthermore, we employ structural connections to segment brain regions, calculating FC for regions with established structural connections in the native space individually. This strategy serves to further curtail superfluous information, fostering heightened accuracy and sensitivity. In a comparable context, previous studies have found that connections between subregion of subcortical nuclei and cortical brain area, derived from structural mapping and segmentation, exhibited greater efficacy in predicting the corresponding function at the individual level. Chen et al.^34^found that the FC of the subregion thalamus (specifically the mediodorsal nucleus) with the prefrontal cortex predicted the treatment improvement, whereas the FC of other thalamic nuclei with cerebral cortex did not predict the treatment efficacy, indicating that subregions within nucleus based on neuronal connections would benefit to the precision of therapeutic interventions.

As anticipated, our findings revealed that subject‐specific CBTC circuits outperformed conventional atlas‐based CBTC connections when using an SVM classifier to distinguish stroke patients from healthy controls. Additionally, we confirmed that positive/combined connections within individualized CBTC circuits mapping significantly predicted variations in UE‐FMA scores, whereas conventional atlas‐based FC values within CBTC circuits had no significant predictive impact on motor behavioral scores in the CPM model. It is also important to note that in our study, negative (inhibition) connections did not independently predict UE‐FMA scores, which may be related to the fact that very few of the patients we included were in the acute phase of stroke. In the early poststroke period, particularly reduced inhibition has been linked to increased neuronal plasticity and functional recovery. In contrast, during the recovery and chronic phases, the activation of the ipsilesional (affected side) brain is more strongly associated with better outcomes.^35^, ^36^ Importantly, the CPM model pinpointed essential connections within brain circuits, offering valuable intervention targets for poststroke upper limb motor recovery.

Numerous studies had demonstrated that poststroke patients undergo structural and functional reorganizations, closely linked to motor recovery.^37^, ^38^, ^39^, ^40^ Technically, neuroimaging has become crucial for tracking and correlating these changes with behavioral improvements. The main challenge in rehabilitation is identifying specific neural circuits for functions and optimizing their engagement and modification.^41^ Through CPM, we pinpointed CBTC connections correlated with upper limb motor function, suggesting targeted rehabilitation to enhance motor recovery. Capitalizing on individual‐specific CBTC connections, we can customize individualized rehabilitation plans aimed at selectively stimulating and enhancing connections relevant to upper limb motor function. Specifically, we can choose highly correlated connections for ccPAS to further optimize motor recovery.^5^, ^42^ Besides, these findings inspire pharmaceutical strategies targeting specific CBTC connections to supplement motor recovery. This introduces a fresh avenue within rehabilitation medicine, enabling more precise localization and facilitation of neural plasticity to promote motor function rehabilitation.

Previous studies supported the effectiveness of targeting specific brain areas in stroke patients, like the M1^43^ and DLPFC.^44^ However, it is important to acknowledge that functional performance relies on the intricate interplay of multiple brain regions. Hence, single‐target stimulation has certain limitations and may not fully harness the potential of neural plasticity from the perspective of neural circuits.

The connection patterns within the CBTC circuits based on individual‐level VCPs‐based segmentation present promising targets for neuromodulation, which could potentially provide more precise and effective approaches for stroke patient rehabilitation. Selectively stimulating compromised connections within CBTC circuits can bolster adaptive neuroplasticity and promote motor function recovery. For instance, utilizing the connection contributing to motor function prediction as potential targets for brain modulation, with the aim of enhancing its connectivity, might hold the potential to result in more favorable clinical rehabilitation outcomes when compared with conventional single‐target stimulation approaches. To modulate the critical connection, paired TMS intervention can be employed to boost brain connectivity by capitalizing on Hebbian plasticity mechanisms. This method involves precise targeting of cortical mapping points associated with subcortical nuclei, thus enabling the selective enhancement of cortical–subcortical connections.^45^

Additionally, these neuromodulation strategies can be individualized based on an individual's specific CBTC circuits connection patterns, thereby achieving more precise modulation effects. Our study underscores the inherent interindividual disparities and diversity within the CBTC circuits, aligning with the contemporary emphasis on personalized treatment within the field of rehabilitation medicine. The insights derived from our study offer valuable guidance for the evolution of future neural modulation strategies. By amalgamating individualized CBTC circuits connection insights, we can develop more precise and effective treatment methodologies for poststroke motor rehabilitation, thereby opening new avenues for the future of neural rehabilitation research.

### 3.1 Limitation

Several limitations warrant consideration. First, due to our interest in predicting poststroke motor function, we defined a set of a priori regions of interest (ROIs) of CBTC circuits using the standard atlas. Future studies could examine a different parcellation, such as whole‐brain parcellations, given the importance of other brain functional components (e.g., cognitive demands) for motor rehabilitation.^46^ A second limitation was that we only studied intrahemispherical connectivity in CBTC circuits, while future work could investigate whether interhemispherical connectivity could be jointly studied to better characterize brain functional connections. Third, we did not differentiate stroke types, stages, or the severity of motor function impairment in detail. Thus, testing these results on a larger and longitudinal dataset will be needed in future studies to improve their generalizability. Finally, this study focused on upper limb motor function and utilized a UE‐FMA measure for evaluating motor performance of the affected upper limb in stroke patients. Future studies could focus on lower limb, hand, and other preference dimensions of motor function. Different behavioral measures may identify distinct patterns of discriminative and predictive performance.

## 4 CONCLUSION

In summary, this study has undertaken the integration of structural and functional brain imaging data to mapping the predefined parallel loops of CBTC circuits at an individual level, including the “long” cortico–striato–pallido–thalamo–cortical circuit and the “short” cortico–striato–thalamo–cortical circuit, for studying altered connectivity patterns in the context of poststroke upper motor dysfunction. Employing FC values derived from the individual mapping CBTC circuits based on VCPs‐based segmentation, we have demonstrated the effective differentiation of poststroke patients from the healthy controls. Furthermore, our findings have shown the significant predictive capacity of those FC values in relation to the motor function of poststroke patients, bearing considerable clinical relevance. Specifically, targeting highly correlated connections using noninvasive neuromodulation techniques can further optimize motor recovery. These findings offer valuable insights with the potential to make substantial contributions to poststroke motor rehabilitation and the development of innovative neuromodulation strategies in the future.

## 5 MATERIALS AND METHODS

### 5.1 Participants

For the discovery dataset, a total of 128 participants, including 64 stroke patients and 64 healthy controls, were recruited from the Yueyang Hospital of Integrated Traditional Chinese and Western Medicine, Shanghai University of Traditional Chinese Medicine. For the external validation, we collected data from 31 stroke patients and 31 age‐ and gender‐matched healthy controls at the Shanghai Panoramic Medical Imaging Diagnostic Center. Inclusion criteria were as follows: (1) the patient had a diagnosis of hemorrhagic or ischemic stroke, (2) age between 30 and 80 years, (3) onset within 1 year, (4) presence of unilateral upper limb motor deficit, specifically classified as moderate to serve. Exclusion criteria were: (1) bi‐hemispheric hemorrhagic or ischemic strokes; (2) concurrent cognitive impairments or aphasia; (3) any contraindication to MRI (e.g., pacemaker). The age‐ and gender‐matched healthy participants without contraindication to MRI were served as a comparison group.

All participants gave informed written consent before entering the study. The study protocol had been approved by the local ethics committee at the Yueyang Hospital of Integrated Traditional Chinese and Western Medicine, Shanghai University of Traditional Chinese Medicine, and which was carried out under the Declaration of Helsinki.

### 5.2 Motor performance measurement

The UE‐FMA was used for evaluating motor performance of the affected upper limb in the included poststroke patients, consisting of 33 terms. Scores on the UE‐FMA range from 0 to 66, with higher scores denoting better motor function.^47^

### 5.3 MRI data processing and lesion identification

All DICOM images were converted to NIFTI using dcm2niix (https://github.com/rordenlab/dcm2niix). In order to pool right and left lesion patient together to improve statistical power, imaging data from 15 patients with lesions on left hemisphere were flipped along the mid sagittal line. So that, for all patients, we defined left side as the contra‐lesioned hemisphere, and right side as the ipsilesioned hemisphere.^40^ We used the automated lesion identification toolkit within Statistical Parametric Mapping software (SPM12: Wellcome Trust Centre for Neuroimaging, https://www.fil.ion.ucl.ac.uk/spm/) to derive lesion images. Image acquisition parameters and all the specific data preprocessing steps were provided in Supporting Information.

### 5.4 Preparation for ROIs in CBTC circuits

**FIGURE 4:**
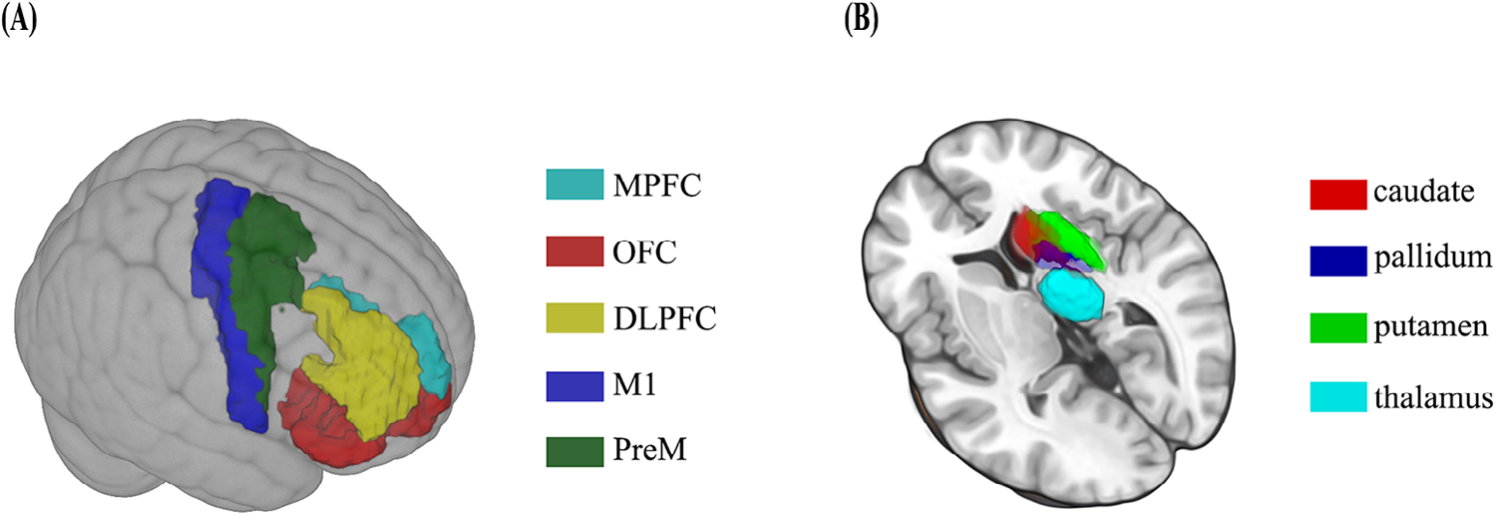
The cortical (A) and subcortical (B) atlas‐defined regions of interest.

In this study, we focused on the CBTC circuits which played central roles in motor control and feedback. First, the ROIs in the standard MNI space was obtained from the standard atlas. For the cortical ROIs, we identified MPFC, DLPFC, M1, premotor cortex, and orbitofrontal cortex with reference to the brainnetome atlas with 246 subdivisions (BN246)^12^, ^48^ (Table S6). For the subcortical ROIs of the CBTC circuits, we employed the automated anatomical labeling template to define caudate, putamen, pallidum, and thalamus (Figure 4).

### 5.5 Mapping the individual parallel loops of CBTC circuits and FC calculation in the native space

#### 5.5.1 Probabilistic tracking and VCPs‐based segmentation

Probabilistic diffusion tractography and VCPs‐based segmentation was performed in native diffusion space using the previously described probabilistic index of connectivity (PICo) algorithm implemented in the freely available Camino software package (http://www.cs.ucl.ac.uk/research/medic/camino/).^12^, ^49^ Definition of seeds and targets and the process of generating PICo maps were provided in the Supporting Information. Finally, two cortico–basal ganglia loops consisting of five distinct cortical areas projecting to the subcortical structures were mapped in each hemisphere: the “long” cortico–striato–pallido–thalamo–cortical loop and the “short” cortico–striato–thalamo–cortical loop. Detailed information of the specific “short” loop (consisting of 19 connections each hemisphere) (Figure S12) and “long” loop (consisting of 21 connections each hemisphere) (Figure S13) can be found in Supporting Information (Table S7).

#### 5.5.2 FC calculation of CBTC circuits connections

Before calculating FC, the cortical regions and the subregions of the subcortical structures acquired from the above segmentation were warped from the diffusion space to the native BOLD space using FLIRT. In addition, the BOLD data were preprocessed the same as those above, with the exception of normalization to the standard MNI space. Subsequently, FC values were calculated between pairwise ROIs of each connection within the two predefined CBTC loops in the native BOLD space, then converted to *z* values using Fisher's *r*‐to‐*z* transformation to improve normality for further analysis. Finally, 40 FC values within CBTC circuits were obtained in each hemisphere (Table S7 and Figures S12 and S13).

#### 5.5.3 Conventional atlas‐based FC calculation without subcortical segmentation

As a comparison, we conducted conventional atlas‐based FC analysis of resting‐state fMRI data in the standard MNI space treating every subcortical structure as an undivided whole. Using a ROI approach, FC calculation was performed between ROIs via Pearson correlation analysis in MNI space (correlations between ROI time series) and converted to *z* values using Fisher's *r*‐to‐*z* transformation to improve normality for further analysis. In this study, we focused on the cortico–subcortical projective connections and the connections between subcortical areas in CBTC circuits. Finally, 26 FC values were obtained in each hemisphere, involving 20 connections between five cortical ROIs and four subcortical ROIs and six connections between each pair of four subcortical ROIs.

### 5.6 Classification by SVM

Machine learning classification algorithms have been shown to be reliable and valid with FC data.^50^, ^51^ Among these algorithms, SVM stands out as an efficient method for classification, finding broad applications in disease diagnosis or medical assistance. It excels when dealing with machine learning datasets that involve a limited number of samples but a substantial number of features.^31^ In this study, we used the Libsvm tools (https://www.csie.ntu.edu.tw/~cjlin/libsvm/) to conduct the SVM classification, discriminating poststroke patients from healthy controls.^34^ The classification normally consists of two phases: training and testing. In the training phase, the SVM identifies a decision boundary called “hyperplane” in the input feature space that separates the data. In the testing phase, the trained function is used to predict the class label of a new, previously unseen, test sample data. A binary label with 1 for stroke patients and −1 for healthy controls was used here. Leave‐one‐out‐cross‐validation (LOOCV) was used to evaluate the performance of the SVM classifier.^32^ Two classifiers were established by featuring FC values of connections within the CBTC circuits generated by the two methods. Sensitivity (SEN), specificity (SPE), accuracy (ACC), and AUC were used to evaluate the classifier performance based on the LOOCV results. Detailed information regarding SVM parameter settings and modeling steps is provided in the Supporting Information.

### 5.7 Connectome‐based predictive modeling

CPM, a recently developed method introduced by Shen et al.,^52^ has been extended to develop predictive models for brain‐behavior relationships on functional connections. It enables the estimation of individual differences in connectivity strength to predict a given behavioral measure and employs the strength of those connections to predict behavior in novel individuals. Notably, the connections were separated into positive connections (FC values were positively correlated with behavioral scores) and negative connections (FC values were negatively correlated with behavioral scores). Detailed steps for establishing the CPM model can be found in the Supporting Information. To predict the UE‐FMA scores, we developed three distinct linear regression models: one for positive connections, another for negative connections, and a combined model that encompassed all connections. We utilized a LOOCV approach, where each participant's predicted value, that is, the “left‐out” participant, was generated iteratively using data from all other participants as the training dataset, until all participants had their predicted values computed. The model's predictive performance was evaluated by correlating the predicted values and observed behavioral scores using the Spearman correlation (*r* value).^53^

### 5.8 Statistical analysis

SPSS 24.0 software (SPSS, Chicago, IL, USA) was used for statistical analyses of demographic data and FC data. For age data that followed a normal distribution, a two‐sample *t*‐test was used. For age data that did not follow a normal distribution, the Mann–Whitney *U* test was applied. Gender differences were assessed using chi‐squared tests (significance level: *p* < 0.05). To assess group differences in FC values after *z*‐transformation derived from two different methods, two‐sample *t*‐tests were performed. FDR correction was applied to account for multiple comparisons, maintaining the adjusted threshold for significance at *p* < 0.05.

To assess the statistical significance of our classification and prediction models, we employed permutation tests. For SVM analysis, 5000 random permutations of the class labels. The classifiers were repeatedly trained and evaluated to generate a null distribution of AUC values. The *p* value was calculated as the proportion of sampled permutations that yielded AUC values greater than or equal to the AUC obtained from the original data. For CPM analysis, permutation testing was done by preserving patients’ connectivity values, but while randomly shuffling patients’ UE‐FMA scores 5000 times. The LOOCV prediction procedure was employed to derive empirical null distributions of the correlation coefficients (*r* value) by correlating the predicted values with the observed UE‐FMA scores. The *p* value was calculated as the proportion of sampled permutations that were greater than or equal to the true prediction correlation coefficient. The *p* value of <0.05 was considered significant.

**FIGURE 5:**
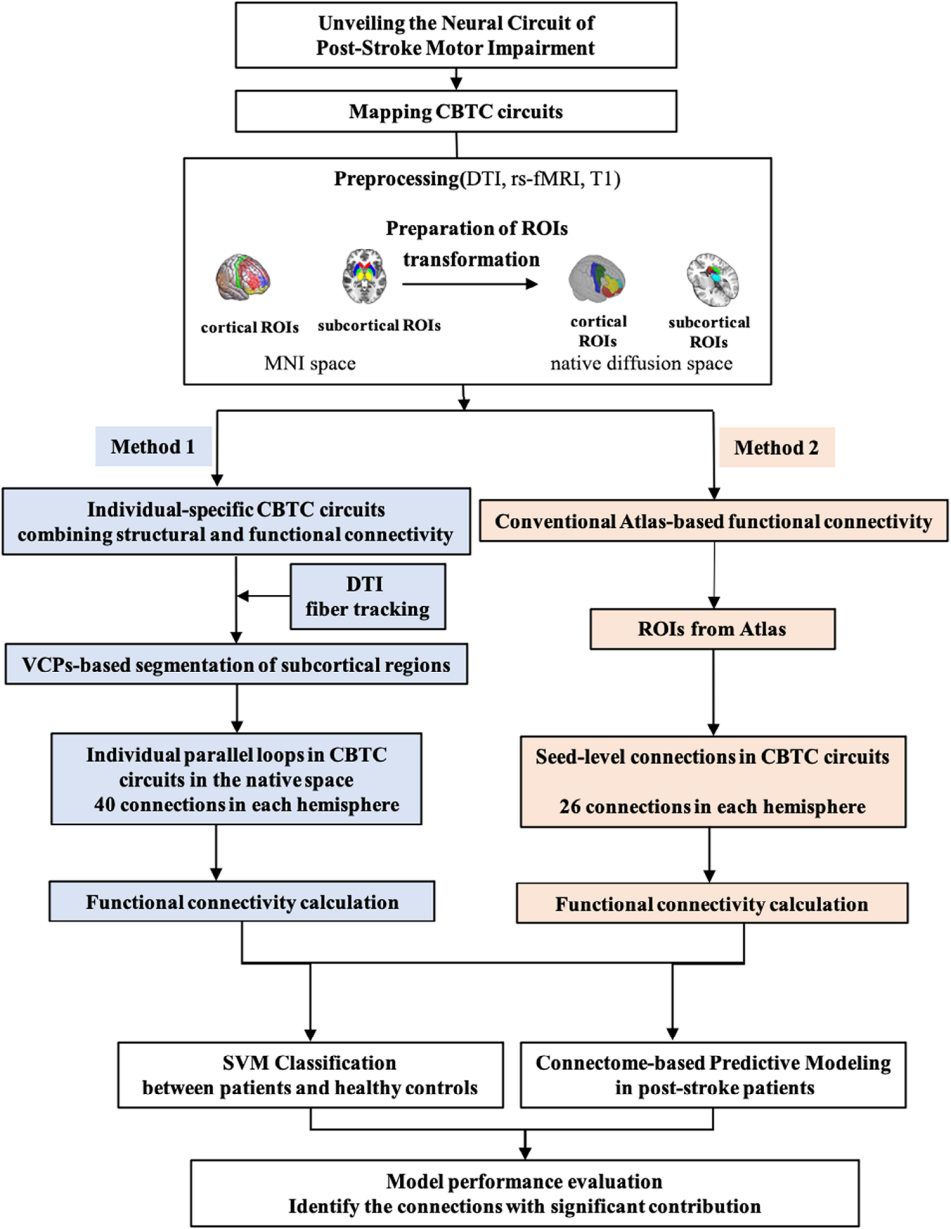
The architecture of the whole pipeline.

The architecture of the whole pipeline is showed in Figure 5.

## AUTHOR CONTRIBUTIONS

*Data analysis and manuscript draft*: Xin Xue and Jia‐Jia Wu. *Data acquisition*: Xiang‐Xin Xing and Jie Ma. *Visualization*: Jun‐Peng Zhang and Yun‐Ting Xiang. *Conception and design of the study*: Mou‐Xiong Zheng, Xu‐Yun Hua, and Jian‐Guang Xu. All authors have read and approved the final manuscript.

## CONFLICT OF INTEREST STATEMENT

The authors disclose no potential conflict of interest.

## ETHICS STATEMENT

The study protocol had been approved by the local ethics committee at the Yueyang Hospital of Integrated Traditional Chinese and Western Medicine, Shanghai University of Traditional Chinese Medicine (NO: KYSKSB2020‐116). Written informed consent was obtained from all participants.

## Supporting information



Supporting Information

## Data Availability

The datasets in this study are available from the corresponding author on reasonable request.
