# Supplementary material for: Mapping individual cortico–basal ganglia–thalamo–cortical circuits integrating structural and functional connectome: implications for upper limb motor impairment poststroke
Source: MedComm (2020). 2024 Oct 6;5(10):e764. doi: 10.1002/mco2.764 (PMC11456677; doi:10.1002/mco2.764)
Supplement: Supplementary file 1 — Supporting Information [file MCO2-5-e764-s001.docx]

**Title：**Mapping individual cortico-basal ganglia-thalamo-cortical circuits integrating structural and functional connectome: implications for upper limb motor impairment post-stroke

**Running head：** Altered CBTC circuits after stroke

Xin Xue, PhD^1#^, Jia-Jia Wu, MD, PhD^1,4*^, Xiang-Xin Xing, MD, PhD^5^, Jie Ma, MD, PhD^1^, Jun-Peng Zhang, PhD^2^, Yun-Ting Xiang, PhD^2^, Mou-Xiong Zheng, MD, PhD^3,4#^, Xu-Yun Hua, MD, PhD^3,4#^, Jian-Guang Xu, MD, PhD^1,2,4*^

1. Department of Rehabilitation Medicine, Yueyang Hospital of Integrated Traditional Chinese and Western Medicine, Shanghai University of Traditional Chinese Medicine, Shanghai, China

2. School of Rehabilitation Science, Shanghai University of Traditional Chinese Medicine, Shanghai, China

3. Department of Traumatology and Orthopedics, Yueyang Hospital of Integrated Traditional Chinese and Western Medicine, Shanghai University of Traditional Chinese Medicine, Shanghai, China

4. Engineering Research Center of Traditional Chinese Medicine Intelligent Rehabilitation, Ministry of Education, Shanghai, China

5. Rehabilitation Center, Qilu Hospital of Shandong University, Jinan, Shandong, China

**^#^ Xin Xue, Mou-Xiong Zheng and Xu-Yun Hua are co-first authors.**

**^*^** **Correspondence to:**

1. Jian-Guang Xu, M.D., Ph.D.

No.1200 Cailun Road, Shanghai, China

Tel: +86 021-51322091

Fax: +86 021-51322042

E-mail: xjg@shutcm.edu.cn

2. Jia-Jia Wu, M.D., Ph.D.

No.110 Ganhe Road, Shanghai, China

Tel: +86 021-51322091

Fax: +86 021-51322042

E-mail: wujiajia@shutcm.edu.cn

**Table S1**

**Demographic and clinical variables in participants with post-stroke.**

| **ID** | **Age** | **Sex** | **Stroke type** | **Disease course** | **Affected hemisphere** | **Lesion location** | **Diabetes mellitus** | **Hypertension** |  |
| --- | --- | --- | --- | --- | --- | --- | --- | --- | --- |
| 1 | 60 | F | Haemorrhagic | 12 | R | Basal ganglia  (subcortical) | Yes | Yes |  |
| 2 | 36 | F | Haemorrhagic | 3 | L | Basal ganglia  (subcortical) | No | Yes |  |
| 3 | 71 | M | Ischemic | 6 | R | Basal ganglia  (subcortical) | Yes | Yes |  |
| 4 | 68 | F | Haemorrhagic | 7 | L | Basal ganglia  (subcortical) | Yes | Yes |  |
| 5 | 67 | M | Ischemic | 5 | L | Basal ganglia  (subcortical) | No | Yes |  |
| 6 | 64 | M | Ischemic | 9 | R | Basal ganglia  (subcortical) | Yes | Yes |  |
| 7 | 73 | F | Ischemic | 11 | R | Basal ganglia  (subcortical) | Yes | Yes |  |
| 8 | 60 | F | Haemorrhagic | 12 | L | Parietal lobe  (cortical) | No | No |  |
| 9 | 72 | M | Ischemic | 6 | L | Basal ganglia  (subcortical) | Yes | Yes |  |
| 10 | 68 | F | Ischemic | 5 | R | Basal ganglia  (subcortical) | Yes | Yes |  |
| 11 | 44 | F | Haemorrhagic | 9 | R | Basal ganglia  (subcortical) | No | No |  |
| 12 | 58 | M | Ischemic | 12 | R | Basal ganglia  (subcortical) | Yes | Yes |  |
| 13 | 64 | F | Ischemic | 11 | R | Basal ganglia  (subcortical) | No | Yes |  |
| 14 | 36 | F | Ischemic | 8 | L |  | Yes | Yes |  |
| 15 | 67 | M | Ischemic | 10 | L | Basal ganglia  (subcortical) | No | Yes |  |
| 16 | 69 | M | Haemorrhagic | 6 | R | Basal ganglia  (subcortical) | Yes | No |  |
| 17 | 64 | M | Ischemic | 9 | R | Basal ganglia  (subcortical) | Yes | Yes |  |
| 18 | 66 | F | Haemorrhagic | 12 | R | Basal ganglia  (subcortical) | Yes | No |  |
| 19 | 50 | F | Ischemic | 12 | L | Basal ganglia  (subcortical) | Yes | No |  |
| 20 | 70 | F | Ischemic | 3 | R | Basal ganglia  (subcortical) | Yes | Yes |  |
| 21 | 70 | F | Ischemic | 2 | L | Basal ganglia  (subcortical) | Yes | Yes |  |
| 22 | 44 | F | Ischemic | 10 | R | Basal ganglia  (subcortical) | No | No |  |
| 23 | 58 | M | Ischemic | 12 | R | Basal ganglia  (subcortical) | No | Yes |  |
| 24 | 47 | F | Ischemic | 1 | L | Basal ganglia  (subcortical) | Yes | Yes |  |
| 25 | 57 | M | Ischemic | 12 | R | Basal ganglia  (subcortical) | Yes | No |  |
| 26 | 73 | F | Haemorrhagic | 7 | R | Basal ganglia  (subcortical) | No | Yes |  |
| 27 | 59 | F | Ischemic | 9 | L | Basal ganglia  (subcortical) | Yes | No |  |
| 28 | 66 | M | Ischemic | 12 | L | Basal ganglia  (subcortical) | Yes | Yes |  |
| 29 | 70 | M | Ischemic | 1 | R | Basal ganglia  (subcortical) | Yes | Yes |  |
| 30 | 67 | F | Ischemic | 12 | L | Basal ganglia  (subcortical) | No | Yes |  |
| 31 | 69 | F | Ischemic | 8 | R | Basal ganglia  (subcortical) | No | Yes |  |
| 32 | 54 | F | Haemorrhagic | 6 | L | Corona radiata  (subcortical) | Yes | Yes |  |
| 33 | 78 | F | Ischemic | 1 | L | Thalamus  (subcortical) | Yes | Yes |  |
| 34 | 75 | F | Ischemic | 6 | R | Basal ganglia  (subcortical) | Yes | No |  |
| 35 | 43 | F | Ischemic | 1 | R | Basal ganglia  (subcortical) | No | Yes |  |
| 36 | 57 | F | Ischemic | 12 | R | Basal ganglia  (subcortical) | Yes | No |  |
| 37 | 65 | M | Ischemic | 4 | R | Basal ganglia  (subcortical) | No | Yes |  |
| 38 | 45 | M | Ischemic | 6 | R | Middle cerebral artery  (subcortical) | Yes | Yes |  |
| 39 | 68 | F | Ischemic | 12 | L | Basal ganglia  (subcortical) | No | Yes |  |
| 40 | 55 | M | Ischemic | 10 | R | Basal ganglia  (subcortical) | Yes | No |  |
| 41 | 41 | M | Ischemic | 12 | R | Basal ganglia  (subcortical) | Yes | Yes |  |
| 42 | 60 | M | Ischemic | 4 | L | Cerebral peduncle, basal ganglia, and corona radiata  (subcortical) | No | Yes |  |
| 43 | 64 | M | Ischemic | 3 | R | Insular cortex and basal ganglia  (subcortical) | No | No |  |
| 44 | 65 | M | Ischemic | 5 | R | Middle cerebral artery  (subcortical) | No | Yes |  |
| 45 | 48 | F | Ischemic | 10 | L | Basal ganglia  (subcortical) | No | Yes |  |
| 46 | 52 | M | Ischemic | 7 | L | Basal ganglia and corona radiata  (subcortical) | Yes | Yes |  |
| 47 | 58 | M | Ischemic | 12 | L | Basal ganglia  (subcortical) | No | No |  |
| 48 | 40 | M | Ischemic | 12 | L | Basal ganglia  (subcortical) | No | No |  |
| 49 | 41 | M | Haemorrhagic | 11 | R | Basal ganglia and corona radiata  (subcortical) | No | Yes | |
| 50 | 59 | M | Ischemic | 3 | L | Brain stem  (subcortical) | Yes | Yes | |
| 51 | 65 | F | Ischemic | 9 | R | Basal ganglia  (subcortical) | No | No | |
| 52 | 67 | M | Ischemic | 3 | R | Middle cerebral artery  (subcortical) | Yes | Yes | |
| 53 | 43 | M | Haemorrhagic | 3 | R | Basal ganglia  (subcortical) | No | Yes | |
| 54 | 44 | M | Haemorrhagic | 3 | L | Frontal cortex, temporal cortex and occipital cortex  (cortical) | No | No | |
| 55 | 48 | M | Haemorrhagic | 3 | L | Basal ganglia  (subcortical) | No | Yes | |
| 56 | 55 | F | Ischemic | 11 | L | Temporal cortex (cortical), insular cortex, and basal ganglia (subcortical) | No | No | |
| 57 | 40 | M | Ischemic | 5 | L | Paraventricular and basal ganglia  (subcortical) | Yes | Yes | |
| 58 | 42 | M | Ischemic | 12 | R | Middle cerebral artery  (subcortical) | Yes | Yes | |
| 59 | 50 | M | Haemorrhagic | 6 | L | Paraventricular and basal ganglia  (subcortical) | No | Yes | |
| 60 | 54 | F | Ischemic | 3 | L | Frontal cortex and parietal cortex  (cortical) | No | Yes | |
| 61 | 43 | M | Haemorrhagic | 11 | R | Basal ganglia  (subcortical) | No | Yes | |
| 62 | 45 | M | Haemorrhagic | 6 | R | Basal ganglia  (subcortical) | No | Yes | |
| 63 | 58 | M | Ischemic | 8 | L | Paraventricular  (subcortical) | No | Yes | |
| 64 | 65 | M | Ischemic | 4 | L | Pons  (subcortical) | No | No | |

**Table S2**

**Demographic and Clinical Characteristics of enrolled participants in the external validation sample.**

| **Characteristic** | **Stroke patients**  **(n = 31)** | **Healthy controls**  **(n = 31)** | ***t / χ²*** | ***p*-value** |
| --- | --- | --- | --- | --- |
| Age (yr) ^*^ | 64.3 ± 7.891 | 60.8 ± 9.226 | 1.598 | 0.115 |
| Gender (male / female) ^†^ | 21 / 10 | 15 / 16 | 2.385 | 0.123 |
| Diabetes mellitus, n (%) ^†^ | 20 (64.5) | -- |  | -- |
| Hypertension, n (%) ^†^ | 23 (74.2) | -- |  | -- |
| Stroke type (ischaemic / haemorrhagic) ^†^ | 22 / 9 | -- |  | -- |
| Disease course (month) ^#^ | 7 (7) | -- |  | -- |
| UE-FMA ^#^ | 17 (40) | -- |  | -- |

Data are expressed as the mean ± SD (*), median (interquartile range, IQR) (^#^), number (†) or percentage. UE-FMA, The Upper Extremity Fugl-Meyer Assessment.

**Table S3**

**Demographic and clinical variables of post-stroke patients in the external validation sample.**

| **ID** | **Age** | **Sex** | **Stroke type** | **Disease course** | **Affected hemisphere** | **Lesion location** | **Diabetes mellitus** | **Hypertension** |  |
| --- | --- | --- | --- | --- | --- | --- | --- | --- | --- |
| 1 | 57 | F | Haemorrhagic | 11 | L | Basal ganglia, thalamus and corona radiata  (subcortical) | Yes | Yes |  |
| 2 | 60 | F | Ischemic | 3 | L | Basal ganglia  (subcortical) | Yes | Yes |  |
| 3 | 60 | M | Ischemic | 1.5 | R | Paraventricular (subcortical) | Yes | No |  |
| 4 | 71 | M | Haemorrhagic | 7 | L | Basal ganglia  (subcortical) | Yes | Yes |  |
| 5 | 51 | M | Haemorrhagic | 9 | L | Basal ganglia  (subcortical) | No | Yes |  |
| 6 | 58 | M | Ischemic | 9 | R | Basal ganglia  (subcortical) | Yes | Yes |  |
| 7 | 69 | M | Haemorrhagic | 9 | L | Basal ganglia  (subcortical) | Yes | Yes |  |
| 8 | 64 | M | Haemorrhagic | 12 | L | Parietal lobe  (cortical) | No | Yes |  |
| 9 | 65 | M | Ischemic | 1.5 | L | Basal ganglia  (subcortical) | Yes | Yes |  |
| 10 | 78 | M | Ischemic | 5 | R | Basal ganglia  (subcortical) | Yes | Yes |  |
| 11 | 73 | M | Ischemic | 4 | R | Basal ganglia  (subcortical) | No | Yes |  |
| 12 | 54s | M | Ischemic | 12 | R | Basal ganglia  (subcortical) | No | Yes |  |
| 13 | 61 | M | Ischemic | 1.5 | R | Basal ganglia  (subcortical) | No | Yes |  |
| 14 | 68 | F | Ischemic | 8 | L | Basal ganglia  (subcortical) | Yes | Yes |  |
| 15 | 70 | M | Ischemic | 12 | R | Basal ganglia  (subcortical) | No | Yes |  |
| 16 | 72 | M | Haemorrhagic | 6 | R | Basal ganglia  (subcortical) | Yes | No |  |
| 17 | 71 | M | Ischemic | 5 | R | Corona radiata (subcortical) | Yes | Yes |  |
| 18 | 60 | F | Haemorrhagic | 12 | R | Basal ganglia  (subcortical) | Yes | No |  |
| 19 | 48 | M | Ischemic | 9 | L | Basal ganglia  (subcortical) | Yes | No |  |
| 20 | 73 | M | Ischemic | 3 | L | Basal ganglia  (subcortical) | Yes | Yes |  |
| 21 | 65 | M | Ischemic | 5 | L | Thalamus  (subcortical) | Yes | Yes |  |
| 22 | 67 | M | Ischemic | 10 | R | Basal ganglia  (subcortical) | No | No |  |
| 23 | 52 | F | Haemorrhagic | 2.5 | R | Basal ganglia  (subcortical) | No | Yes |  |
| 24 | 68 | F | Ischemic | 1 | L | Basal ganglia  (subcortical) | Yes | Yes |  |
| 25 | 51 | M | Ischemic | 1.5 | R | Basal ganglia  (subcortical) | Yes | No |  |
| 26 | 75 | F | Haemorrhagic | 10 | L | Basal ganglia  (subcortical) | No | Yes |  |
| 27 | 60 | F | Ischemic | 2 | R | Basal ganglia  (subcortical) | Yes | No |  |
| 28 | 67 | M | Ischemic | 8 | R | Basal ganglia  (subcortical) | Yes | Yes |  |
| 29 | 62 | F | Ischemic | 12 | R | Basal ganglia  (subcortical) | Yes | Yes |  |
| 30 | 68 | M | Ischemic | 12 | R | Basal ganglia  (subcortical) | No | Yes |  |
| 31 | 74 | F | Ischemic | 3 | R | Basal ganglia  (subcortical) | No | No | |

**Table S4**

**Group differences in functional connectivity in CBTC circuits** **based on probabilistic tracking and voxel connectivity profiles-based segmentation in the external validation sample.**

|  |  | Hemisphere | ROI1 | ROI2 | *p*-FDR value |
| --- | --- | --- | --- | --- | --- |
| Patients < Healthy controls | | |  |  |  |
|  | “long” loop | affected | caudate_M1_ | M1 | 0.036 |
|  | “long” loop | affected | putamen_M1_ | M1 | 0.004 |
|  | “short” loop | affected | putamen_DLPFC_ | DLPFC | 0.036 |
|  | “short” loop | affected | putamen_M1_ | M1 | 0.036 |

Subcortical area with subscript referred to the specific sub-division of the subcortical area connected with the subscript cortical area as determined through diffusion white matter fiber tractography. M1, primary motor cortex. DLPFC, dorsolateral prefrontal cortex.

**Table S5**

**Group differences in functional connectivity in CBTC circuits** **based on probabilistic tracking and voxel connectivity profiles-based segmentation in** **ischemic stroke patients and healthy controls (the discovery dataset).**

|  |  | Hemisphere | ROI1 | ROI2 | *p-*FDR value |
| --- | --- | --- | --- | --- | --- |
| Patients < Healthy controls | | |  |  |  |
|  | “long” loop | affected | caudate_M1_ | M1 | <0.001 |
|  | “short” loop | affected | caudate_M1_ | M1 | 0.009 |

Subcortical area with subscript referred to the specific sub-division of the subcortical area connected with the subscript cortical area as determined through diffusion white matter fiber tractography. M1, primary motor cortex.

**Table S****6**

**Cortical regions of interest (ROIs) in the CBTC circuits obtained from Brainnetome Atlas.**

| Brain region | Abbreviation | Atlas label (L) | Atlas label (R) | Brodmann |
| --- | --- | --- | --- | --- |
| Primary motor cortex | M1 | 53 | 54 | area 4 (head and face region) |
|  |  | 57 | 58 | area 4 (upper limb region) |
|  |  | 59 | 60 | area 4 (trunk region) |
|  |  | 61 | 62 | area 4 (tongue and larynx region) |
| Premotor cortex | PreM | 7 | 8 | dorsolateral area 6 |
|  |  | 9 | 10 | medial area 6 |
|  |  | 25 | 26 | ventrolateral area 6 |
|  |  | 55 | 56 | caudal dorsolateral area 6 |
|  |  | 63 | 64 | caudal ventrolateral area 6 |
| Dorsolateral prefrontal cortex | DLPFC | 15 | 16 | dorsal area 9/46 |
|  |  | 19 | 20 | area 46 |
|  |  | 21 | 22 | ventral area 9/46 |
| Medial prefrontal cortex | MPFC | 13 | 14 | medial area 10 |
|  |  | 177 | 178 | rostroventral area 24 |
|  |  | 179 | 180 | pregenual area 32 |
|  |  | 187 | 188 | subgenual area 32 |
| Orbital frontal cortex | OFC | 41 | 42 | medial area 14 |
|  |  | 43 | 44 | orbital area 12/47 |
|  |  | 45 | 46 | lateral area 11 |
|  |  | 47 | 48 | medial area 11 |
|  |  | 49 | 50 | area 13 |
|  |  | 51 | 52 | ateral area 12/47 |

**Table S7**

**Comprehensive overview of 40 connections within the the “long” cortico-striato-pallido-thalamo-cortical loop and the “short” cortico-striato-thalamo-cortical loop of CBTC circuits.**

| “short” loop | ROI1 | ROI2 | “long” loop | ROI1 | ROI2 |
| --- | --- | --- | --- | --- | --- |
| 1 | Caudate _DLPFC_ _&_ _thalamus_ | DLPFC | 20 | Caudate _DLPFC_ _&_ _pallidum_ | DLPFC |
| 2 | Caudate _OFC_ _&_ _thalamus_ | OFC | 21 | Caudate _OFC_ _&_ _pallidum_ | OFC |
| 3 | Caudate _M1 &_ _thalamus_ | M1 | 22 | Caudate _M1_ _&_ _pallidum_ | M1 |
| 4 | Caudate _MPFC_ _&_ _thalamus_ | MPFC | 23 | Caudate _MPFC_ _&_ _pallidum_ | MPFC |
| 5 | Caudate _PreM_ _&_ _thalamus_ | PreM | 24 | Caudate _PreM_ _&_ _pallidum_ | PreM |
| 6 | Putamen _DLPFC_ _&_ _thalamus_ | DLPFC | 25 | Caudate _cortex_ _&_ _pallidum_ | Pallidum |
| 7 | Putamen _OFC_ _&_ _thalamus_ | OFC | 26 | Putamen _DLPFC_ _&_ _pallidum_ | DLPFC |
| 8 | Putamen _M1_ _&_ _thalamus_ | M1 | 27 | Putamen _OFC_ _&_ _pallidum_ | OFC |
| 9 | Putamen _MPFC_ _&_ _thalamus_ | MPFC | 28 | Putamen _M1_ _&_ _pallidum_ | M1 |
| 10 | Putamen _PreM_ _&_ _thalamus_ | PreM | 29 | Putamen _MPFC_ _&_ _pallidum_ | MPFC |
| 11 | Thalamus _DLPFC_ _&_ _caudate_ _&_ _putamen_ | DLPFC | 30 | Putamen _PreM_ _&_ _pallidum_ | PreM |
| 12 | Thalamus _OFC_ _& caudate &_ _putamen_ | OFC | 31 | Putamen _cortex_ _&_ _pallidum_ | Pallidum |
| 13 | Thalamus _M1 & caudate & putamen_ | M1 | 32 | Thalamus _DLPFC_ _&_ _pallidum_ | DLPFC |
| 14 | Thalamus _MPFC & caudate &_ _putamen_ | MPFC | 33 | Thalamus _OFC_ _&_ _pallidum_ | OFC |
| 15 | Thalamus _PreM & caudate & putamen_ | PreM | 34 | Thalamus _M1_ _&_ _pallidum_ | M1 |
| 16 | Thalamus _cortex & caudate & putamen_ | Caudate | 35 | Thalamus _MPFC_ _&_ _pallidum_ | MPFC |
| 17 | Thalamus _cortex & caudate & putamen_ | Putamen | 36 | Thalamus _PreM_ _&_ _pallidum_ | PreM |
| 18 | Caudate _cortex_ _&_ _thalamus_ | Thalamus | 37 | Thalamus _cortex_ _&_ _pallidum_ | Pallidum |
| 19 | Putamen _cortex & thalamus_ | Thalamus | 38 | Pallidum _caudate & thalamus & putamen_ | Caudate |
|  |  |  | 39 | Pallidum _caudate & thalamus & putamen_ | Thalamus |
|  |  |  | 40 | Pallidum _caudate & thalamus & putamen_ | Putamen |

Subcortical area with subscripts referred to specific sub-division of the subcortical area that is connected with all the subscript areas as determined through diffusion white matter fiber tractography. For example, caudate _cortex_ _&_ _thalamus_, representing the interconnected junction between cortex-connected and thalamus-connected subdivisions within the caudate nucleu.


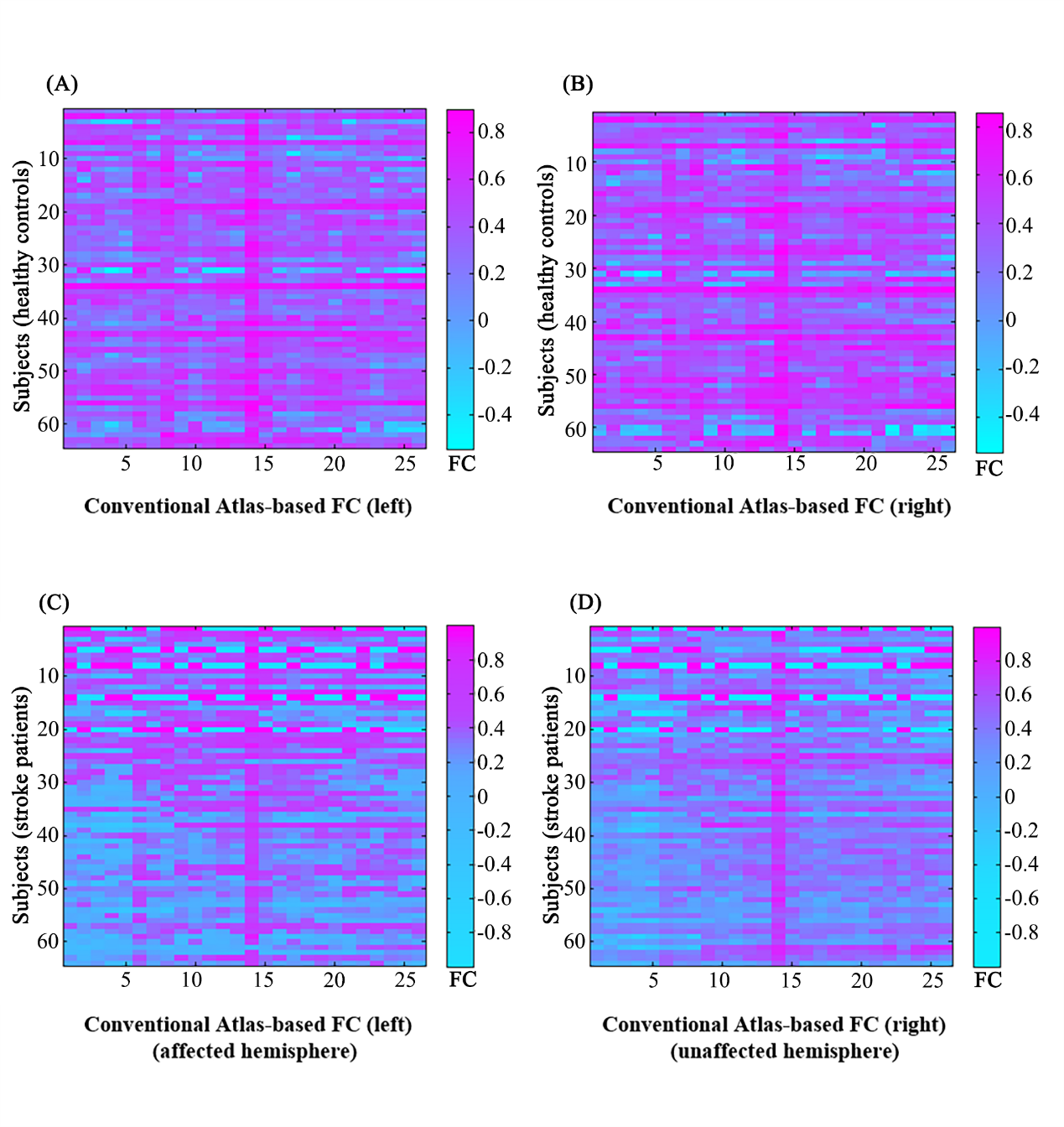


**Figure S1.** The atlas-based functional connectivity (FC) of each participant (26 connections in each hemisphere). (A) FC values of left hemisphere in healthy controls; (B) FC values of right hemisphere in healthy controls; (C) FC values of the affected hemisphere in stroke patients; (D) FC values of the unaffected hemisphere in stroke patients.


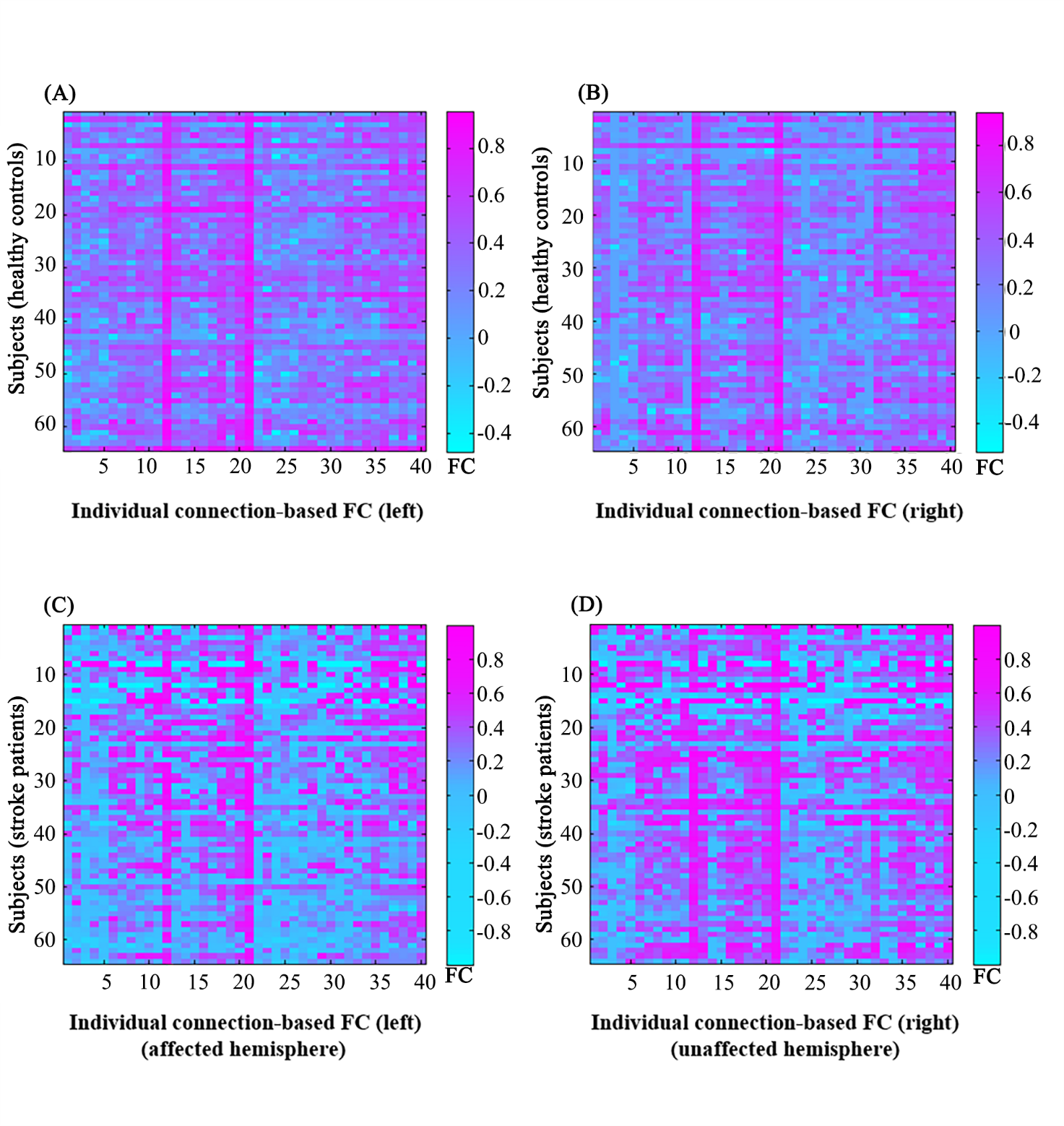


**Figure S2.** The individual connectivity-based functional connectivity (FC) values of each participant (40 connections in each hemisphere). (A) FC values of left hemisphere in healthy controls; (B) FC values of right hemisphere in healthy controls; (C) FC values of the affected hemisphere in stroke patients; (D) FC values of the unaffected hemisphere in stroke patients.


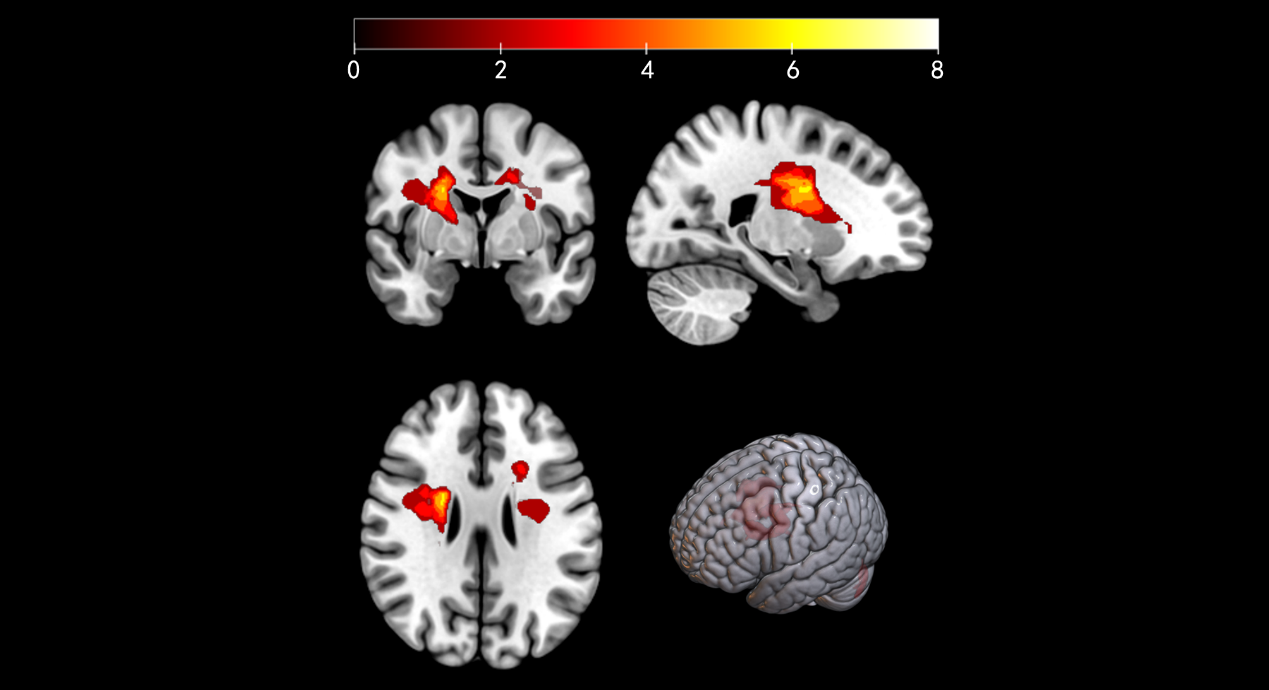


**Figure S3.** Distribution of stroke lesions in the external validation dataset. The color scale represents number of participants with lesioned voxel as evaluated by T1 images.


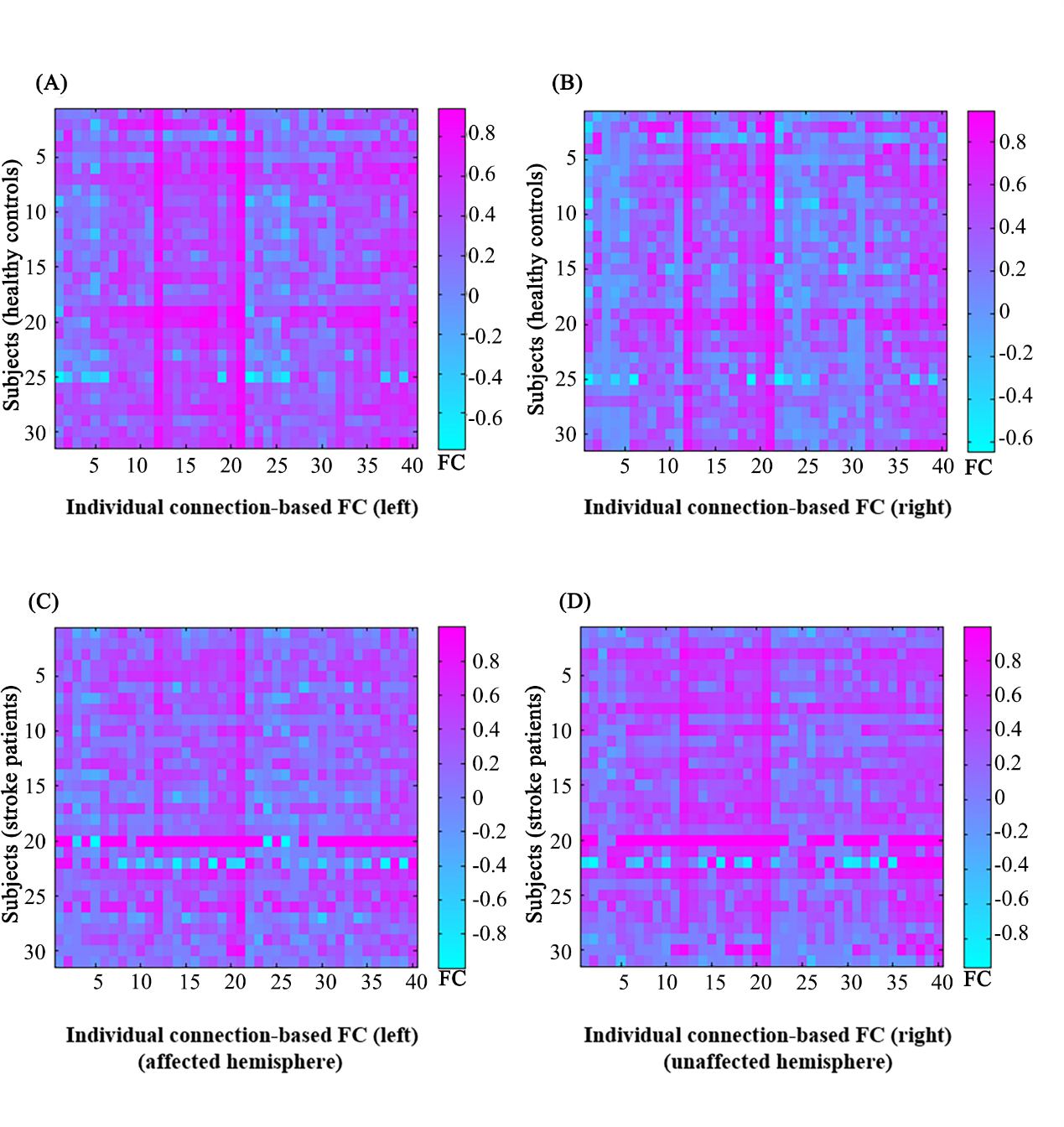


**Figure S4.** The individual connectivity-based functional connectivity (FC) values of each participant (40 connections in each hemisphere) in the external validation sample. (A) FC values of left hemisphere in healthy controls; (B) FC values of right hemisphere in healthy controls; (C) FC values of the affected hemisphere in stroke patients; (D) FC values of the unaffected hemisphere in stroke patients.


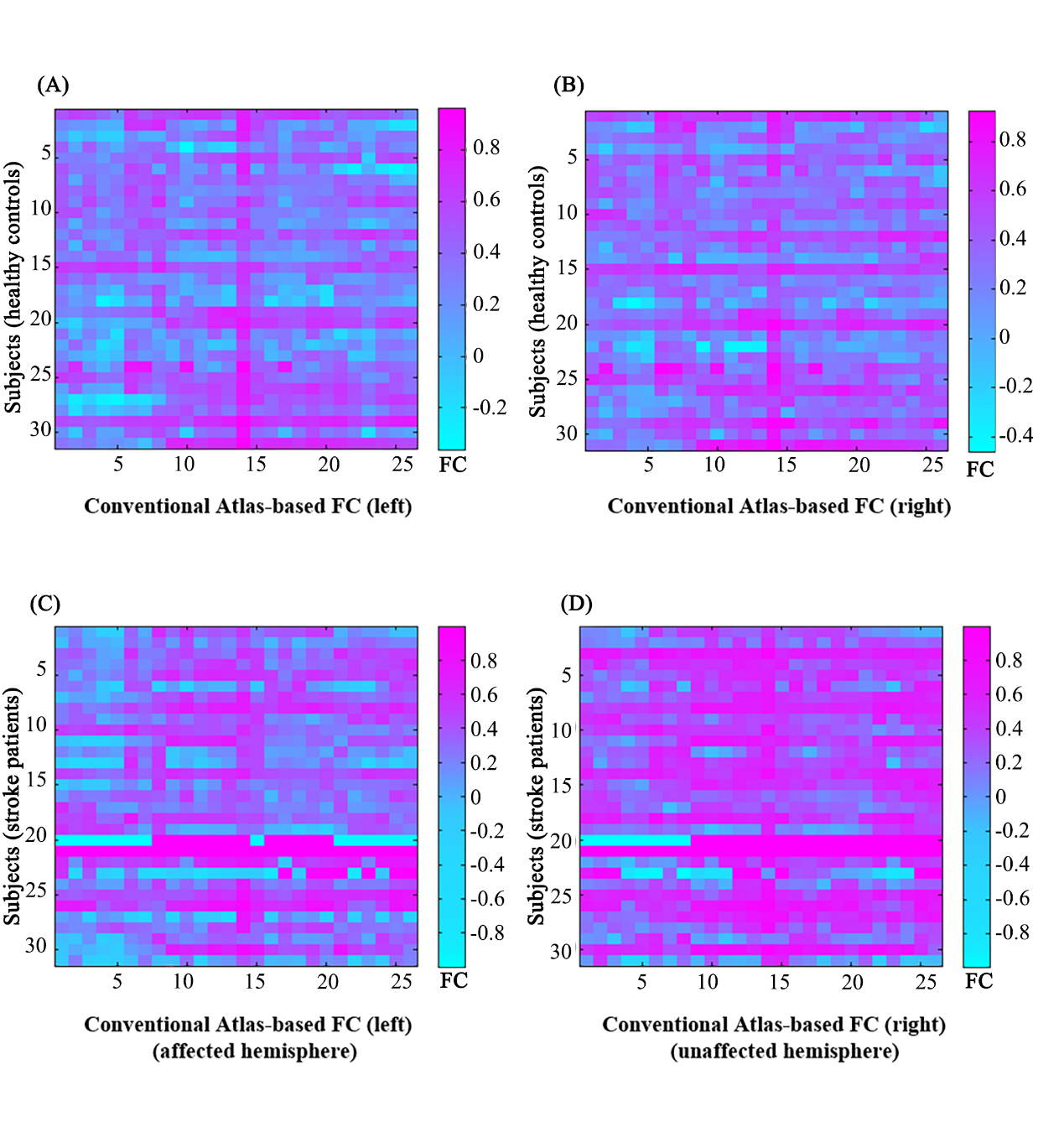


**Figure S5.** The Atlas-based functional connectivity of each participant (26 connections in each hemisphere) in the external validation sample. (A) FC values of left hemisphere in healthy controls; (B) FC values of right hemisphere in healthy controls; (C) FC values of the affected hemisphere in stroke patients; (D) FC values of the unaffected hemisphere in stroke patients.


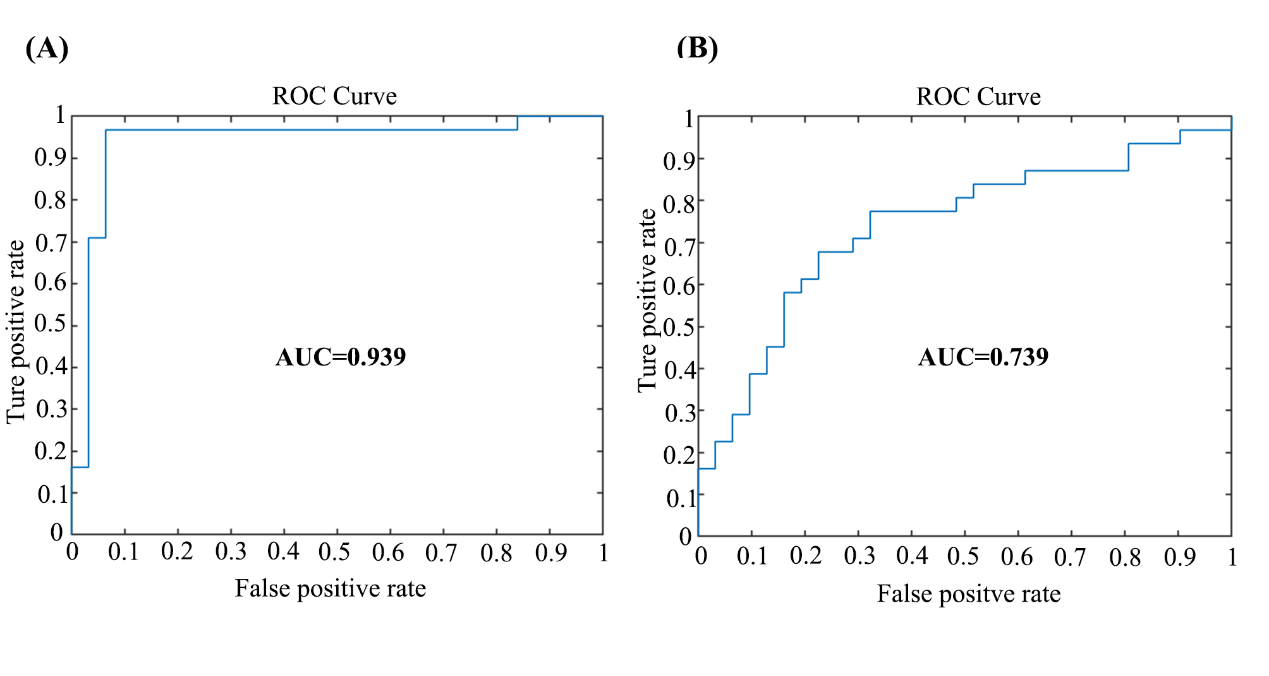


**Figure S6.** Classification performance of SVM using connections derived from the two CBTC circuits mapping methods in the external validation sample. Receiver operator characteristic (ROC) curve of the classifier for the individual mapping CBTC circuits (A) and for the conventional atlas-based CBTC circuits (B).


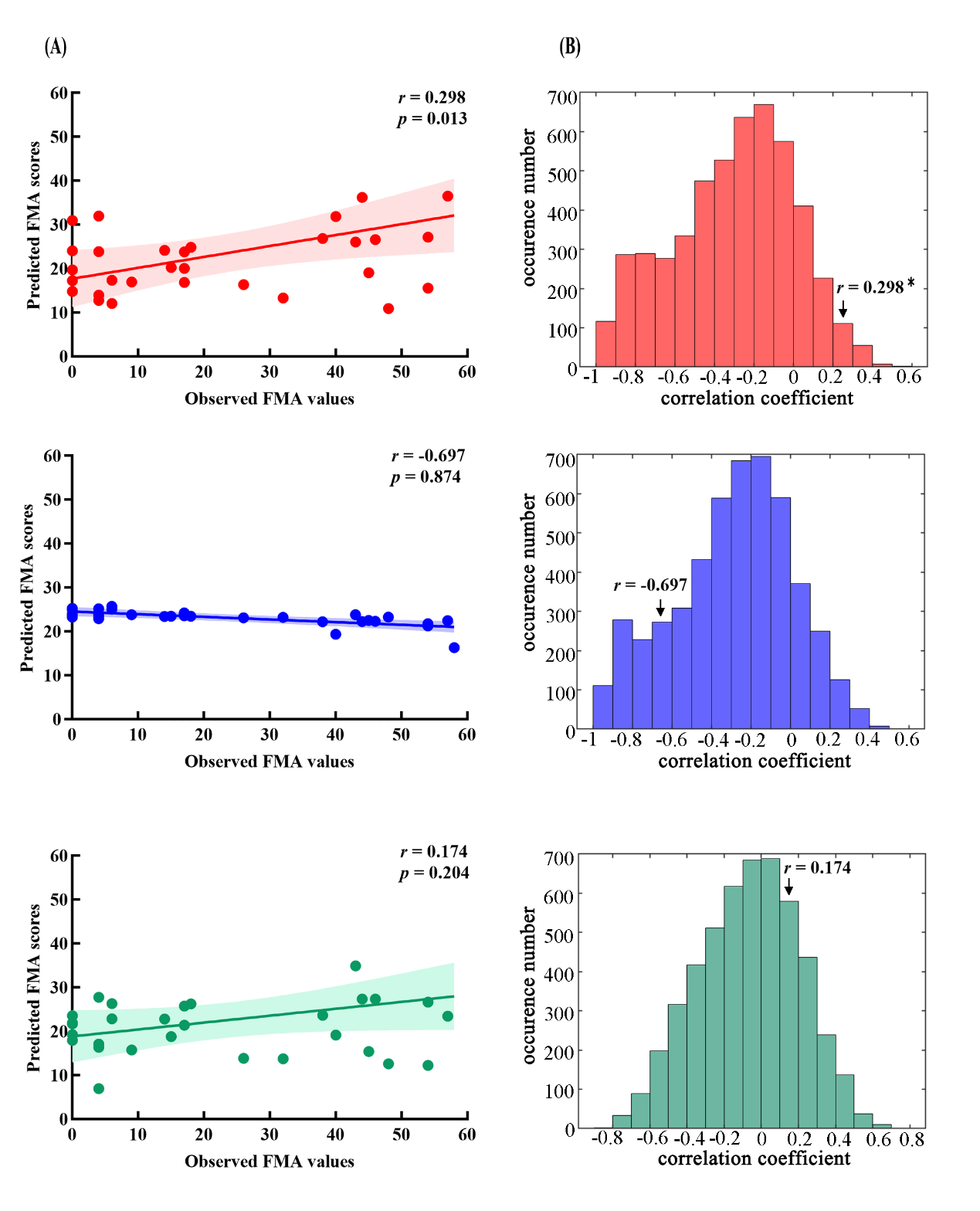


**Figure S7.** Prediction performance of CPM using connections derived from the individual-mapping CBTC circuits based on VCP-based segmentation in the external validation sample. (A) Correlation between observed and predicted UE-FMA scores in positive (red), negative (blue), and combined (green) connections. (B) The distribution of correlation coefficients by a permutation test of 5000 times. **p* < 0.05.


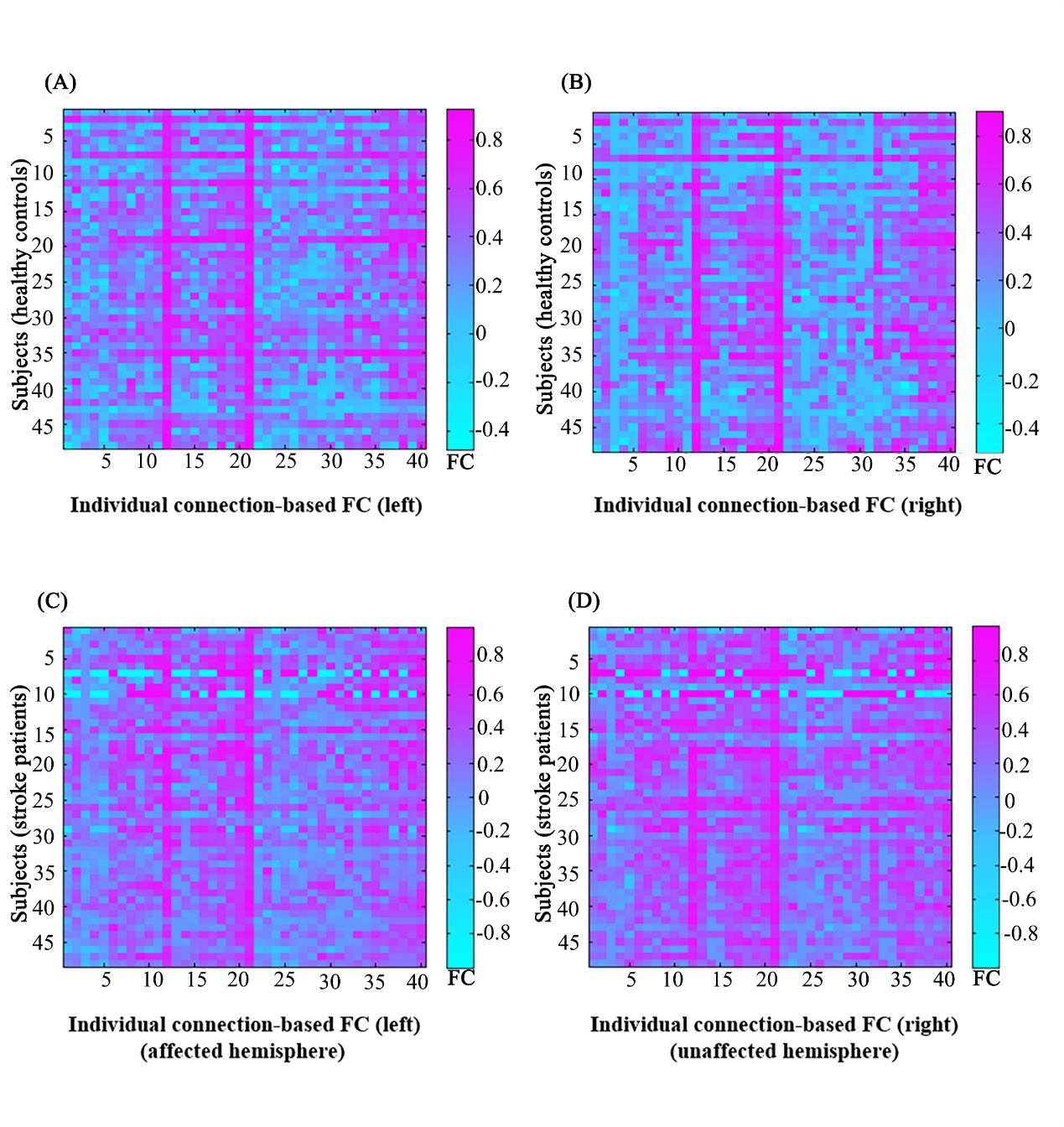


**Figure S8.** The individual connectivity-based functional connectivity (FC) values of each participant (40 connections in each hemisphere) in the ischemic stroke patients (the discovery dataset). (A) FC values of left hemisphere in healthy controls; (B) FC values of right hemisphere in healthy controls; (C) FC values of the affected hemisphere in stroke patients; (D) FC values of the unaffected hemisphere in stroke patients.


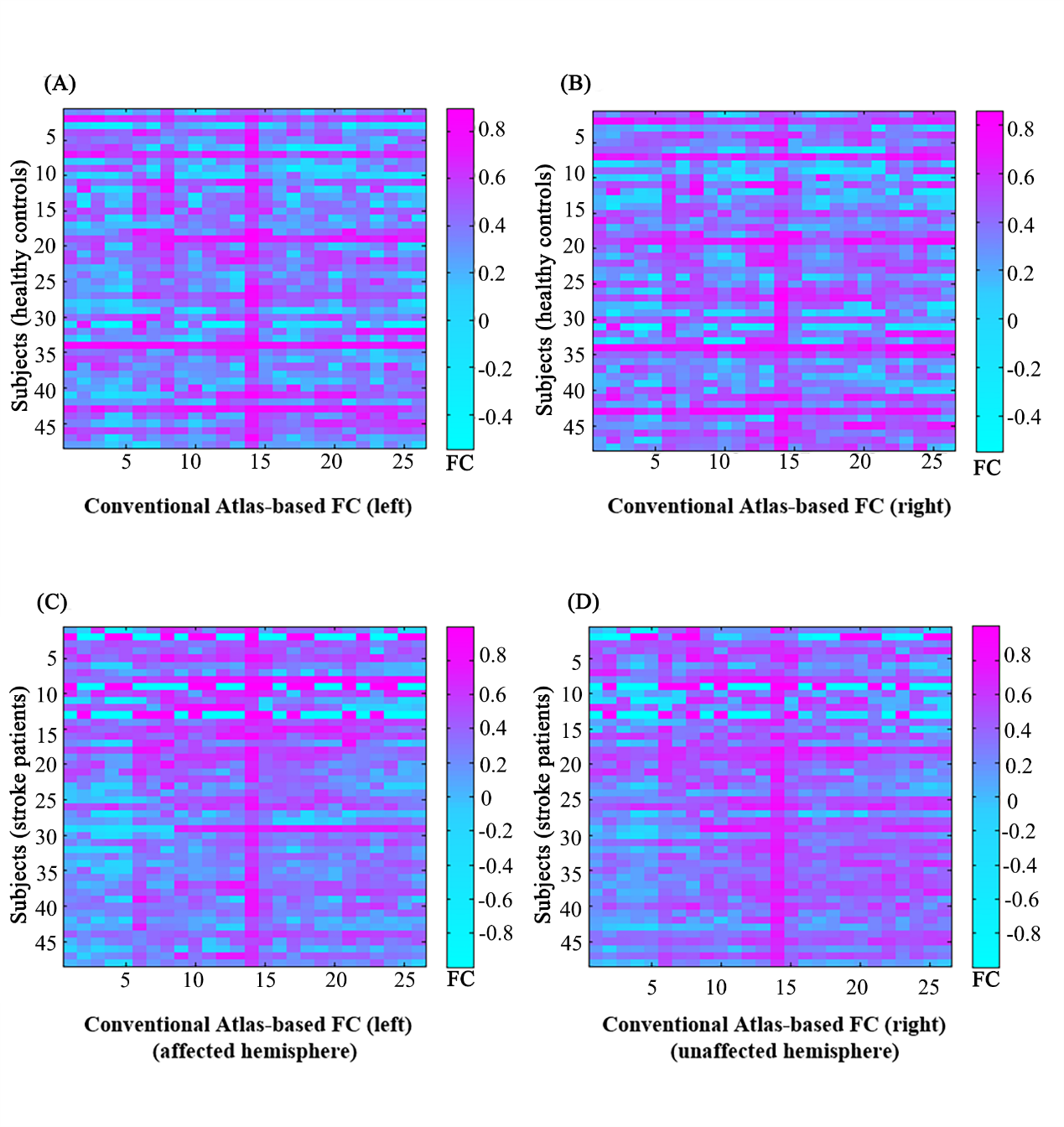


**Figure S9.** The Atlas-based functional connectivity of each participant (26 connections in each hemisphere) in the ischemic stroke patients (the discovery dataset). (A) FC values of left hemisphere in healthy controls; (B) FC values of right hemisphere in healthy controls; (C) FC values of the affected hemisphere in stroke patients; (D) FC values of the unaffected hemisphere in stroke patients.


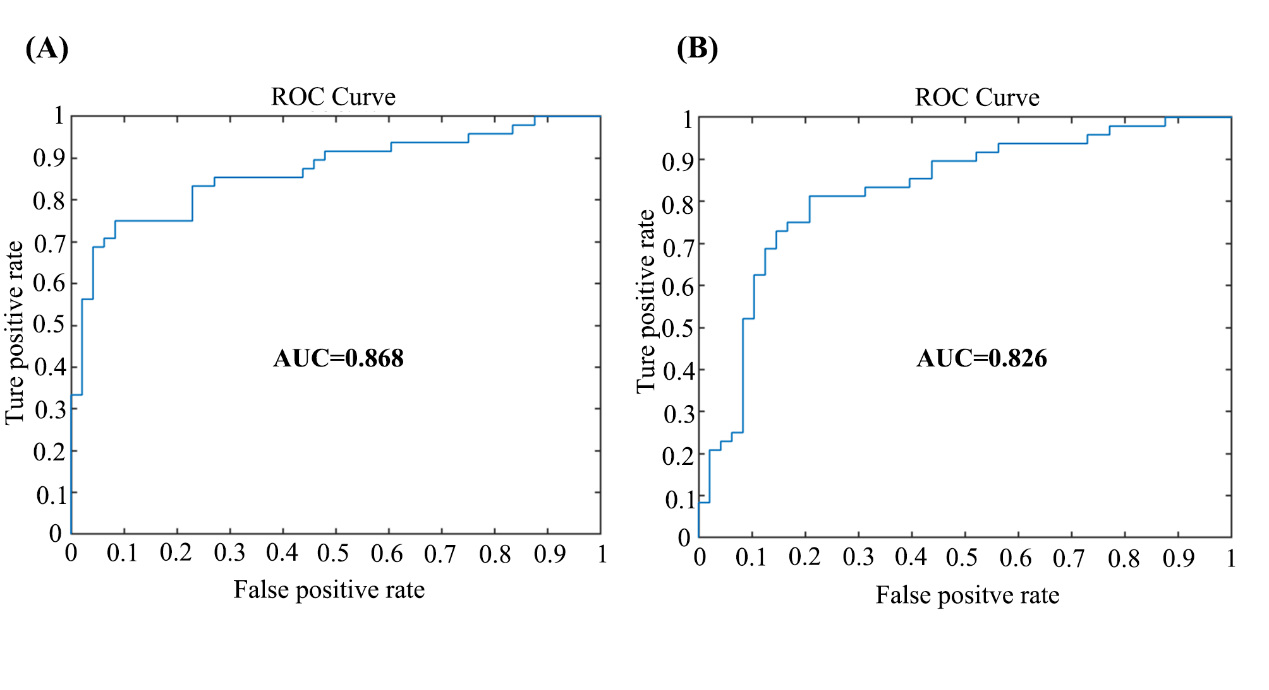


**Figure S10.** Classification performance of SVM using connections derived from the two CBTC circuits mapping methods in the ischemic stroke patients (the discovery dataset). ROC curve of the classifier for the individual mapping CBTC circuits (A) and for the conventional atlas-based CBTC circuits (B).


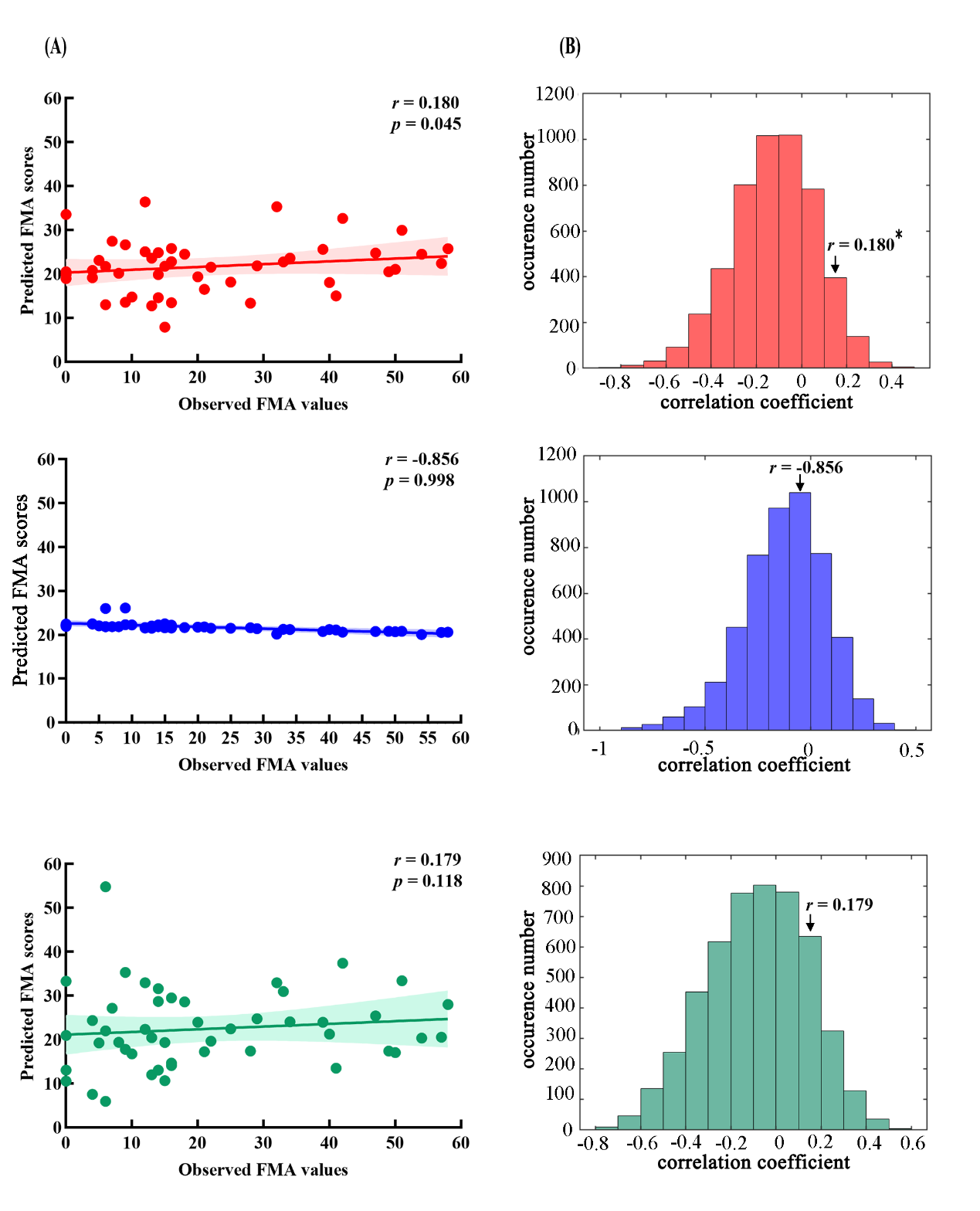


**Figure S11.** Prediction performance of CPM using connections derived from the individual-mapping CBTC circuits based on VCP-based segmentation in the ischemic stroke patients (the discovery dataset). (A) Correlation between observed and predicted UE-FMA scores in positive (red), negative (blue), and combined (green) connections. (B) The distribution of correlation coefficients by a permutation test of 5000 times. **p* < 0.05.


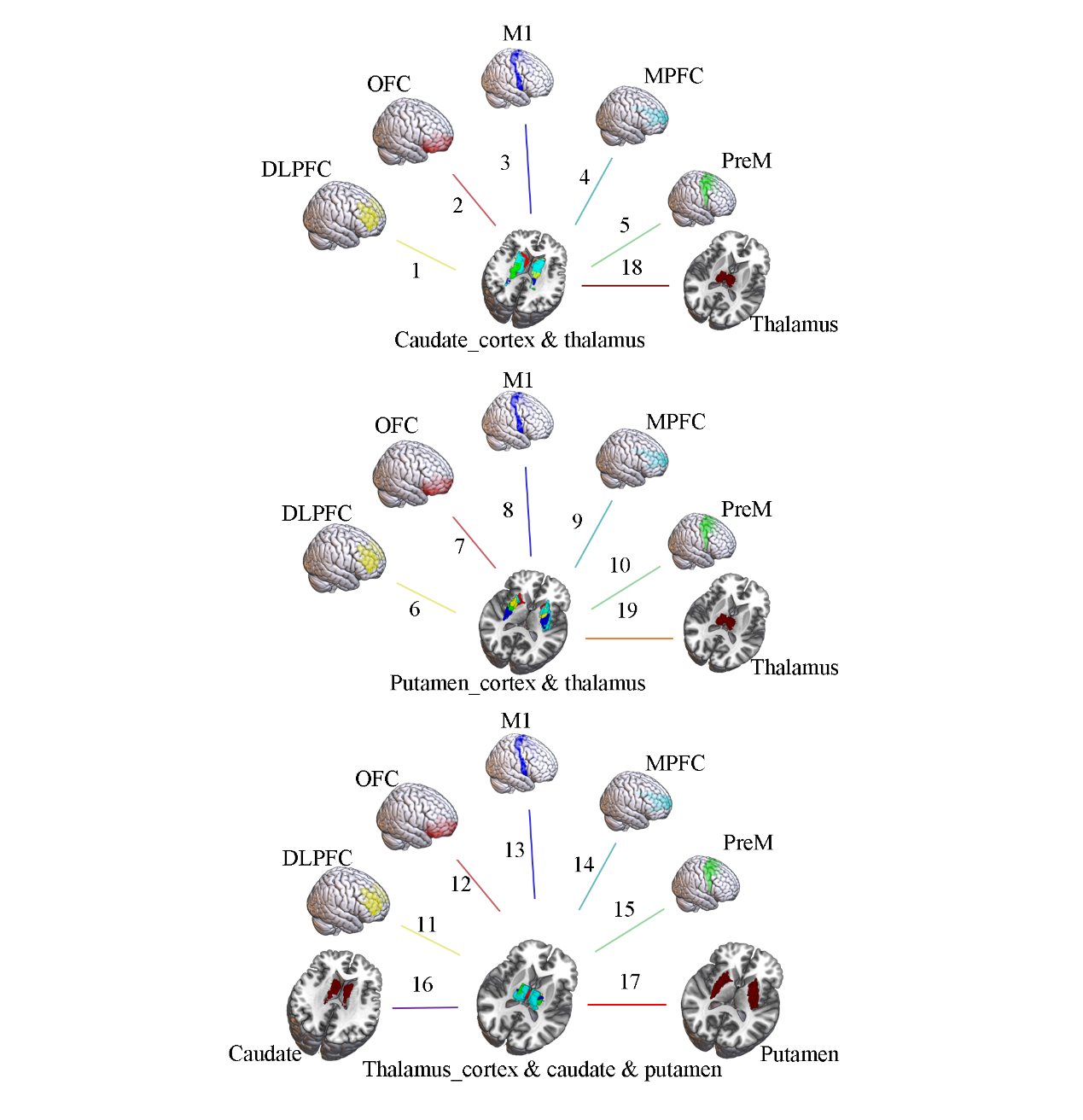


**Figure S12.** Visualizing 19 connections in the “short” cortico – striato – thalamo - cortical loop.


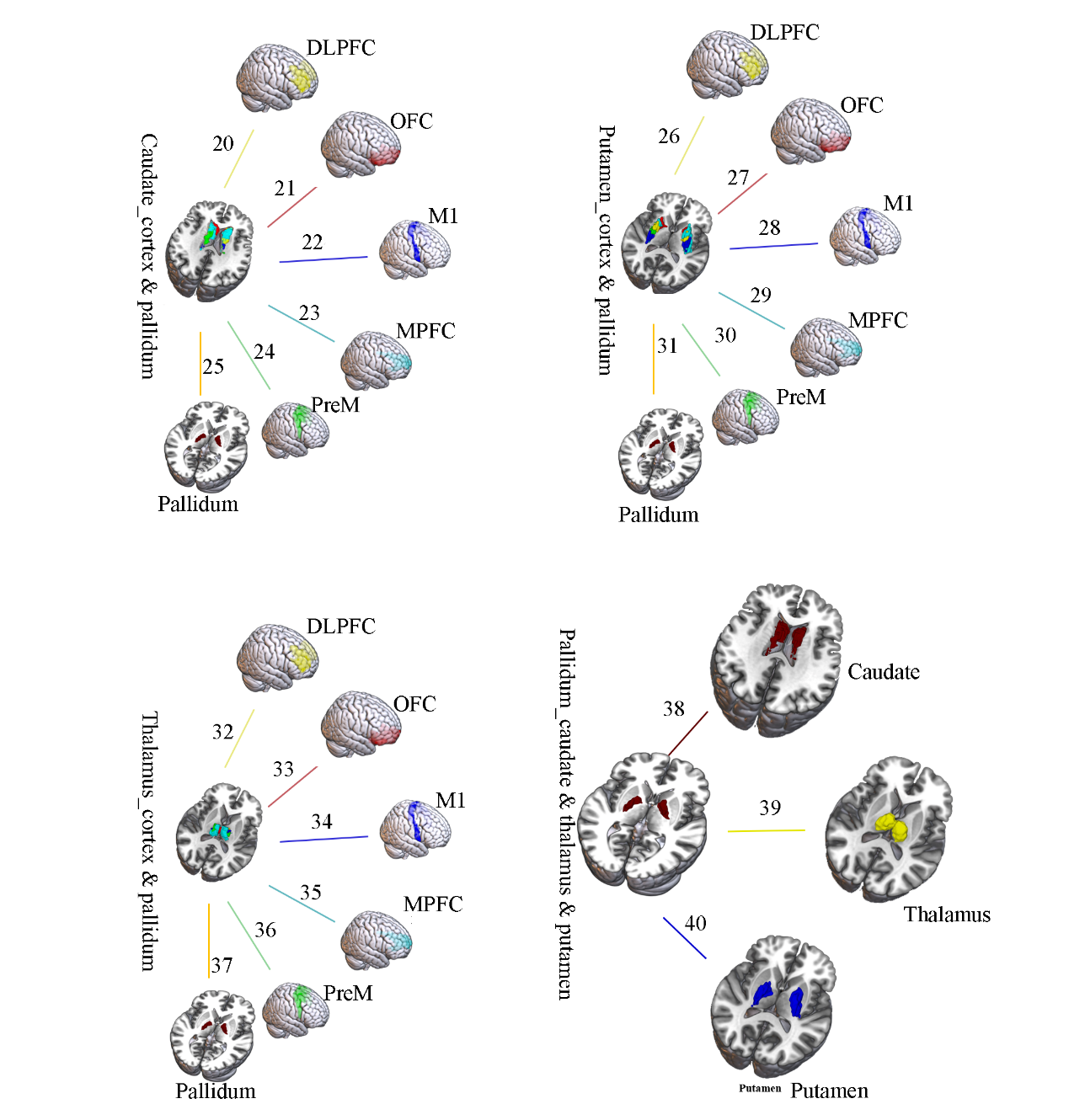


**Figure S13.** Visualizing 21 connections in the “long” cortico – striato – pallido – thalamo - cortical loop.

MRI scan acquisition

All participants underwent multimodal MRI scans (including 3D T1-weighted sagittal scan, DTI scans with b = 1000 s/mm^2^, and resting-state fMRI [rsfMRI] scans) on a 3.0 Tesla MRI system (Verio, Siemens Healthcare, Germany) in Yueyang Hospital of Integrated Traditional Chinese and Western Medicine, Shanghai University of Traditional Chinese Medicine, Shanghai, China. The participants were asked to remain motionless, keep their eyes closed, and not think of anything. Resting-state fMRI images were obtained by using an echo-planar imaging sequence with the following parameters: repetition time (TR) / echo time (TE) = 3000/30 ms, field of view (FOV) = 230 × 230 mm^2^ , matrix size = 64 × 64, slice thickness = 3 mm, 43 axial slices, voxel size = 3.6 × 3.6 × 3 mm^3^, flip angle (FA) = 90°, and a total of 200 volumes. The T1 structural images were acquired using the following parameters: TR / TE = 1900 / 2.93 ms, FOV = 256 × 256 mm^2^, slice thickness = 1 mm, flip angle = 9°, matrix size = 256 × 256, and voxel size = 1 × 1 × 1 mm^3^. The diffusion tensor images (DTI) were obtained using the following parameters: TR / TE = 10000 / 89 ms, slice thickness = 2 mm, flip angle = 90°, matrix size = 128 × 128, two b0 (b = 0) images, diffusion directions = 60 and b value = 1000 s/mm^2^.

The external validation dataset was sourced from the Shanghai Universal Cloud Medical Imaging Diagnostic Center, Shanghai, China. All participants underwent multimodal MRI scans, including 3D T1-weighted sagittal scans, diffusion tensor imaging (DTI) with a b-value of 1000 s/mm², and resting-state functional MRI (rsfMRI), performed on a 3.0 Tesla MRI system (Skyra, Siemens Healthcare, Germany). Resting-state fMRI images were obtained by using an echo-planar imaging sequence with the following parameters: repetition time (TR) / echo time (TE) = 3000/30 ms, field of view (FOV) = 230 × 230 mm^2^ , matrix size = 64 × 64, slice thickness = 3 mm, 45 axial slices, voxel size = 3.6 × 3.6 × 3 mm^3^, flip angle (FA) = 90°, and a total of 210 volumes. The T1 structural images were acquired using the following parameters: TR / TE = 1900 / 2.98 ms, FOV = 160 × 196 mm^2^, slice thickness = 1 mm, flip angle = 9°, matrix size = 160 × 196, and voxel size = 1 × 1 × 1 mm^3^. The diffusion tensor images (DTI) were obtained using the following parameters: TR / TE = 5000 / 82 ms, slice thickness = 2 mm, flip angle = 90°, matrix size = 110 × 110, single b0 (b = 0) images, diffusion directions = 64 and b value = 1000 s/mm^2^.

MRI data preprocessing and Lesion identification

All DICOM images were converted to NIFTI using dcm2niix (https://github.com/rordenlab/dcm2niix). In order to pool right and left lesion patient together to improve statistical power, imaging data from 15 patients with lesions on left hemisphere were flipped along the mid sagittal line. So that, for all patients, we defined left side as the contra-lesioned hemisphere, and right side as the ipsi-lesioned hemisphere ^1^.

Preprocessing of the functional and T1 images was performed using the Data Processing & Analysis of Brain Imaging toolkit (DPABI, <http://rfmri.org/dpabi>) ^2^. The detailed steps of rs-fMRI data preprocessing was as follows: The first 10 time points were removed for magnetic field stabilization. Slice timing correction, head motion correction, and spatial normalization to the Montreal Neurological Institute (MNI) template were performed. Then the normalized images were smoothed with a Gaussian kernel (full-width at half maximum (FWHM)). Covariates were regressed for each voxel, including the white matter (WM) signal, the cerebrospinal fluid signal (CSF), and the Friston 24-parameter model. Additionally, linear detrending, bandpass filter (0.01–0.08 Hz) and nuisance regression (Friston’s 24 motion parameters, the white matter signal, the cerebrospinal fluid signal and global signal) were applied.

All DTI images were identically preprocessed using the functional magnetic resonance imaging of the brain (FMRIB) software library tool (FSL, version 6.0.0, Oxford, UK) ^3^. These DTI images were corrected for motion and eddy currents by eddy. To account for the rotational component of registration, the b-vector files were compensated after motion correction and prior to calculating the b matrices. A brain mask was defined for each subject on the averaged B0 images using the brain extraction toolbox (bet) ^4^. On this basis, fractional anisotropy (FA) maps were computed.

We use the automated lesion identification toolkit (ALI) within Statistical Parametric Mapping software (SPM12: Wellcome Trust Centre for Neuroimaging, <https://www.fil.ion.ucl.ac.uk/spm/>) to derive lesion images. The ALI runs a unified segmentation-normalization procedure optimized for brain with focal lesion. For each patient, an expert in manual lesion-tracking double-checked and enhanced the binary lesion mask against the normalized T1-weighted anatomical whole-brain volume and the neurologist’s lesion description. Then, the lesion overlap map of all patients was generated, and the lesion volume was estimated.

Definition of seeds and targets

We employed all voxels in the basal ganglia structures, thalamus and five cortical regions (MPFC, DLPFC, M1, PreM and OFC) to determine seeds and targets. The caudate, putamen, pallidum, and thalamus were regarded as separate seed sites. For cortical connectivity analyses, five labeled cortical areas (5 per hemisphere) defined above were identified as probabilistic tracking targets; and the subcortical regions served as seeds. For subcortical connectivity analyses, the basal ganglia structures and thalamus were defined as both seeds and targets. All seed and target ROIs acquired in the standard atlas were transformed into each individual’s native diffusion space by using the inverse transformation of T1w images normalized to the standard space followed by a linear transform to diffusion space using FMRIB’s Linear Image Registration Tool (FLIRT) ^5^. The registration was checked visually to ensure that each image was acceptable for analysis and if necessary, manually corrected for accuracy by an expert.

Probabilistic tracking and voxel connectivity profiles-based segmentation

Probabilistic diffusion tractography and voxel connectivity profiles-based segmentation was performed in native diffusion space using the previously described probabilistic index of connectivity (PICo) algorithm implemented in the freely available Camino software package (<http://www.cs.ucl.ac.uk/research/medic/camino/>) ^6,7^. The PICo maps generated by 5000 iterations of the streamline process employing Monte Carlo algorithms to model inherent uncertainty in the orientation of the primary diffusion direction specified for each voxel ^8,9^. Stopping criteria disallowed physiologically unreasonable streamline curvature (> 80° on the scale of a single voxel) or projects to transit voxels with FA value < 0.1. The fundamental principle underlying PICo involves the calculation of the proportion of samples from a seed voxel that successfully reach a designated target region (Definition of seeds and targets can be found in the supplementary materials). With this groundwork, we constructed voxel-based PICo maps for seed structures in the thalamus and basal ganglia to each cortical and subcortical target region. Then, the PICo map of each seed voxel was binarized (thresholded at a probabilistic connectivity index of > 1%) to generate binary strings of the same length at the voxel level which was defined as voxel connectivity profile (VCP). The degree of connection pattern similarity between the VCPs was determined by their Hamming distance. Within a given structure, all seed voxels that have similar VCPs were labelled as belonging to the same cluster. We identified and labeled caudate, putamen, pallidum, and thalamus VCPs relative to each other (subcortical targets) and to each cortical target respectively. Based on the individual labeled subdivisions of the subcortical structures, we pinpointed the anatomical substrate of known relay nuclei in previously described CBTC circuits (as recognized theoretically and neurophysiologically) ^10^. To do this, we used "intersection graphs" to specify relays as those voxels connecting to all related structures in the predefined circuits. Consequently, two cortico-basal ganglia circuits consisting of 5 distinct cortical areas projecting to the following subcortical structures were mapped in each hemisphere of each subject: the “long” cortico-striato-pallido-thalamo-cortical loop and the “short” cortico-striato-thalamo-cortical loop. Specific “long” loop included the following: cortico-caudate-pallidum connections (caudate connected with both cortex and pallidum), cortico-thalamus-pallidum connections (thalamus connected with both cortex and pallidum), cortico-putamen-pallidum connections (putamen connected with both cortex and pallidum), and pallidum connections to putamen, caudate, and thalamus (pallidum connected with the basal ganglia regions and thalamus). Specific “short” loop included the following: cortico-caudate-thalamus connections (caudate connected with both cortex and thalamus), cortico-putamen-thalamus connections (putamen connected with both cortex and thalamus), and cortico-thalamus-striatal (caudate and putamen) connections (thalamus connected with both cortex and the basal ganglia regions).

Classification by support vector machine

Machine learning classification algorithms have been shown to be reliable and valid with FC data ^11,12^. Among these algorithms, Support Vector Machine (SVM) stands out as an efficient method for classification and has found wide applications in disease diagnosis or medical assistance. A brief description of the SVM classifier is a discriminant function. It excels when dealing with machine learning datasets that involve a limited number of samples but a substantial number of features^13^. In this study, we used the Libsvm tools (<https://www.csie.ntu.edu.tw/~cjlin/libsvm/>) to conduct the SVM classification discriminating post-stroke patients from healthy controls ^14^. By using a linear kernel SVM, we not only extracted the feature weights directly but also mitigated the risk of data overfitting^15^. Hyper-parameters of our SVM models underwent careful tuning through grid search. Within the linear kernel SVM, the primary hyperparameter of interest is the penalty parameter cost (C), which sets the trade-off between permitting misclassifications and minimizing training error. For the hyperparameter optimization, a 5-fold cross-validation procedure was performed to determine the parameters with maximum prediction accuracy and high reproducibility.

In this context, we employed binary labels, assigning “1” to post-stroke patients and “-1” to healthy controls. The classification normally consists of two phases: training and testing. During the training phase, the SVM identifies a decision boundary called “hyperplane” in the input feature space that separates the data. In the testing phase, the trained model predicts the class label of a new, previously unseen, test sample data. Leave-one-out-cross-validation (LOOCV) was used to evaluate the performance of the SVM classifier ^16^. In each LOOCV iteration, one participant was left out as a testing set and the remaining participants as the training set for building the classifier. Sensitivity (SEN), specificity (SPE), accuracy (ACC) and area under the curve (AUC) were used to evaluate the classifier performance based on the results of LOOCV. Since the FC features included in this study were predefined based on the motor-related ROIs selected, feature selection was not conducted before the classifier building. In addition, the F score approach was used to rank features, where a higher F score indicated better discrimination ^17^. As detailed in the Results section, the SVM classifier could achieve the highest accuracy and robust classification power when retaining all the connectivity features for each LOOCV fold.

Connectome-based Predictive Modeling

Connectome-based predictive modeling (CPM), a recently developed method introduced by Shen et al., has been extended to develop predictive models of brain-behavior relationships from functional connections, for which individual differences in connectivity strength predict a given behavioral measure and uses the strength of those connections to predict behavior in novel individuals ^18^. In particular, the connections were separated into the positive CBTC connections (FC values were positively correlated with behavioral scores) and the negative CBTC connections (FC values were negatively correlated with behavioral scores) by Spearman correlation analysis (*p*<0.05).

Then, for each subject, we summed the strengths of the positive connections, the negative connections and all of the connections respectively. Next, we built three linear regression models respectively, namely positive connections, negative connections, and a joint model (including all connections), to predict the UE-FMA scores, with a leave-one-out approach. N-1 samples was considered as the training set and the left-out one was used as the testing set. Each resulting model was used to generate predicted UE-FMA values of the left-out subject. We repeated this procedure so that each left-out participant had a predicted behavioral score. The model's predictive performance was evaluated by correlating the predicted values and observed behavioral scores using the Spearman correlation (*r* value) ^19^.

External validation results

We have collected data from 31 stroke patients and 31 age- and gender- matched healthy controls at the Shanghai Universal Cloud Medical Imaging Diagnostic Center for external validation (details in **Table S2, Table S3**; lesion distribution in **Figure S3**). We employed the same methodology as described in our paper, and the results supported our initial findings. The detailed analysis results are as follows:

(1) For FC analysis after subcortical connectivity-based segmentation, post-stroke patients showed decreased FC between the caudate_M1_ and M1 (*p*-FDR = 0.036) and between the putamen_M1_ and M1 (*p*-FDR = 0.004) in the specific “long” loop, between the putamen_DLPFC_ and DLPFC (*p*-FDR = 0.036) and between the putamen_M1_ and M1 (*p*-FDR = 0.036) in the specific “short” loop within the affected hemisphere **(Table S4)**. However, this difference was not statistically significant for the conventional atlas-based FC. The FC values obtained through two different methods in the external validation sample are displayed in **Figure S4** and **Figure S5**.

(2) For classification performance based on the two CBTC circuits mapping methods, the results showed that the linear SVM classifier achieved the highest accuracy of 95.2 % (*p* < 0.001) with a sensitivity of 96.8 %, and a specificity of 93.6 % when including the connections of individual mapping CBTC circuits based on VCPs-based segmentation. The ROC curve analysis acquired an area under the curve (AUC) of 0.939. For the conventional Atlas-based FC analysis, the SVM model correctly classified 71.0% of the participants at most (*p* = 0.040) when including the connections of conventional CBTC circuits, which had an AUC of 0.739 with a sensitivity of 61.3% and a specificity of 80.7% (**Figure S6**).

(3) For prediction performance based on the two CBTC circuits mapping methods, the positive connections in the individual mapping CBTC circuits significantly predicted individual difference in UE-FMA (*r* = 0.298, *p* = 0.013), while negative and combined connections did not significantly predict individual motor performance (negative connections: *r* = -0.697, *p* = 0.874; combined connections: *r* = 0.174, *p* = 0.204) **(Figure S7)**. The conventional Atlas-based FC values in the CBTC circuits had no significant predictive effect on motor behavioral scores in the CPM model (positive connections: *r* = -0.279, *p* = 0.390; negative connections: *r* = -0.609, *p* = 0.689; combined connections: *r* = 0.105, *p* = 0.265).

Results of subgroup analysis (Ischemic stroke patients)

Our dataset included 48 ischemic stroke patients and 16 hemorrhagic stroke patients (discovery dataset, details in **Table S1**). Given the relatively small number of hemorrhagic stroke patients, we determined that it would not be appropriate to conduct a separate statistical analysis for this subgroup. Therefore, we excluded all hemorrhagic stroke patients and performed a new statistical analysis focusing solely on ischemic stroke patients. The detailed results of this new analysis are as follows:

(1) For FC analysis after subcortical connectivity-based segmentation, post-stroke patients showed decreased FC between the caudateM1 and M1 (*p*-FDR < 0.001) in the specific “long” loop and between the caudate_M1_ and M1 (*p*-FDR = 0.009) in the specific “short” loop within the affected hemisphere **(Table S5)**. However, this difference was not statistically significant for the conventional atlas-based FC (all *p*-FDR > 0.05). The FC values obtained through two different methods in the external validation sample are displayed in **Figure S8** and **Figure S9**.

(2) For classification performance based on the two CBTC circuits mapping methods, the results showed that the linear SVM classifier achieved the highest accuracy of 82.3 % (*p* = 0.005) with a sensitivity of 75.0 %, and a specificity of 89.6 % when including the connections of individual mapping CBTC circuits based on VCPs-based segmentation. The ROC curve analysis acquired an area under the curve (AUC) of 0.868. For the conventional Atlas-based FC analysis, the SVM model correctly classified 78.1% of the participants at most (*p* = 0.002) when including the connections of conventional CBTC circuits, which had an AUC of 0.826 with a sensitivity of 77.1% and a specificity of 79.1% (**Figure S10**).

(3) For prediction performance based on the two CBTC circuits mapping methods, the positive connections in the individual mapping CBTC circuits significantly predicted individual difference in UE-FMA (*r* = 0.180, *p* = 0.045), while negative and combined connections did not significantly predict individual motor performance (negative connections: *r* = -0.856, *p* = 0.998; combined connections: *r* = 0.179, *p* = 0.118) **(Figure S11)**. The conventional Atlas-based FC values in the CBTC circuits had no significant predictive effect on motor behavioral scores in the CPM model (positive connections: *r* = 0.025, *p* = 0.141; negative connections: *r* = -0.503, *p* = 0.876; combined connections: *r* = -0.273, *p* = 0.785).

References

1. Miao P, Wang C, Li P, et al. Altered gray matter volume, cerebral blood flow and functional connectivity in chronic stroke patients. Neuroscience Letters 2018;662:331–338.

2. Yan C-G, Wang X-D, Zuo X-N, Zang Y-F. DPABI: Data Processing & Analysis for (Resting-State) Brain Imaging. Neuroinform 2016;14(3):339–351.

3. Smith SM, Jenkinson M, Woolrich MW, et al. Advances in functional and structural MR image analysis and implementation as FSL. NeuroImage 2004;23:S208–S219.

4. Smith SM. Fast robust automated brain extraction. Hum. Brain Mapp. 2002;17(3):143–155.

5. Jenkinson M, Bannister P, Brady M, Smith S. Improved Optimization for the Robust and Accurate Linear Registration and Motion Correction of Brain Images. NeuroImage 2002;17(2):825–841.

6. Draganski B, Kherif F, Kloppel S, et al. Evidence for Segregated and Integrative Connectivity Patterns in the Human Basal Ganglia. Journal of Neuroscience 2008;28(28):7143–7152.

7. Cook PA, Bai Y, Nedjati-Gilani S, et al. Camino: Open-Source Diffusion-MRI Reconstruction and Processing. [date unknown];1.

8. Alexander DC, Barker GJ, Arridge SR. Detection and modeling of non‐Gaussian apparent diffusion coefficient profiles in human brain data. Magn. Reson. Med. 2002;48(2):331–340.

9. Cook PA, Alexander DC, Parker GJM. Modelling noise-induced fibre-orientation error in diffusion-tensor MRI [Internet]. In: 2004 2nd IEEE International Symposium on Biomedical Imaging: Macro to Nano (IEEE Cat No. 04EX821). Arlington, VA, USA: IEEE; 2004 p. 332–335.[cited 2023 Jul 16 ] Available from: http://ieeexplore.ieee.org/document/1398542/

10. Lehéricy S, Ducros M, Van De Moortele P-F, et al. Diffusion tensor fiber tracking shows distinct corticostriatal circuits in humans: DTI Corticostriatal Fibers. Ann Neurol. 2004;55(4):522–529.

11. Dosenbach NUF, Nardos B, Cohen AL, et al. Prediction of Individual Brain Maturity Using fMRI. 2010;329:6.

12. Mohanty R. Machine Learning Classification to Identify the Stage of Brain-Computer Interface Therapy for Stroke Rehabilitation Using Functional Connectivity. Frontiers in Neuroscience 2018;12:14.

13. Vergun S, Deshpande AS, Meier TB, et al. Characterizing Functional Connectivity Differences in Aging Adults using Machine Learning on Resting State fMRI Data [Internet]. Front. Comput. Neurosci. 2013;7[cited 2022 Jul 22 ] Available from: http://journal.frontiersin.org/article/10.3389/fncom.2013.00038/abstract

14. Chen D, Lei X, Du L, Long Z. Use of machine learning in predicting the efficacy of repetitive transcranial magnetic stimulation on treating depression based on functional and structural thalamo-prefrontal connectivity: A pilot study. Journal of Psychiatric Research 2022;148:88–94.

15. Pereira F, Mitchell T, Botvinick M. Machine learning classifiers and fMRI: A tutorial overview. NeuroImage 2009;45(1):S199–S209.

16. Wee C-Y, Yap P-T, Li W, et al. Enriched white matter connectivity networks for accurate identification of MCI patients. NeuroImage 2011;54(3):1812–1822.

17. Chen Y-W, Lin C-J. Combining SVMs with Various Feature Selection Strategies [Internet]. In: Guyon I, Nikravesh M, Gunn S, Zadeh LA, editors. Feature Extraction. Berlin, Heidelberg: Springer Berlin Heidelberg; 2006 p. 315–324.[cited 2022 Aug 30 ] Available from: http://link.springer.com/10.1007/978-3-540-35488-8_13

18. Shen X, Finn ES, Scheinost D, et al. Using connectome-based predictive modeling to predict individual behavior from brain connectivity. Nat Protoc 2017;12(3):506–518.

19. Rosenberg MD, Finn ES, Scheinost D, et al. A neuromarker of sustained attention from whole-brain functional connectivity. Nat Neurosci 2016;19(1):165–171.
